# A Fc-VEGF chimeric fusion enhances PD-L1 immunotherapy via inducing immune reprogramming and infiltration in the immunosuppressive tumor microenvironment

**DOI:** 10.1007/s00262-022-03255-9

**Published:** 2022-07-27

**Authors:** Cheng-Liang Kuo, Han-Yu Chou, Hui-Wen Lien, Chia-An Yeh, Jing-Rong Wang, Chung-Hsing Chen, Chi-Chen Fan, Chih-Ping Hsu, Ting-Yu Kao, Tai-Ming Ko, Alan Yueh-Luen Lee

**Affiliations:** 1 National Institute of Cancer Research, National Health Research Institutes, Zhunan, Miaoli 35053 Taiwan; 2 Department of Biological Science and Technology, National Yang Ming Chiao Tung University, Hsinchu, 300 Taiwan; 3 Institute of Biomedical Sciences, Academia Sinica, Taipei, 11529 Taiwan; 4 Department of Medical Laboratory Science and Biotechnology, Yuanpei University of Medical Technology, Hsinchu, 300 Taiwan; 5 Department of research and development, Marker Exploration Corporation, Taipei, Taiwan; 6 Graduate Institute of Biomedical Sciences, China Medical University, Taichung, 40402 Taiwan; 7 Department of Life Sciences, College of Life Science, National Central University, Taoyuan, 32031 Taiwan; 8 Department of Biotechnology, College of Life Science, Kaohsiung Medical University, Kaohsiung, 80708 Taiwan

**Keywords:** Angiogenesis, Immune reprogramming, Fc-VEGF fusion, Tumor microenvironment, Anti-PD-L1 immunotherapy

## Abstract

**Background:**

Immunotherapy is an emerging cancer therapy with potential great success; however, immune checkpoint inhibitor (*e.g*., anti-PD-1) has response rates of only 10–30% in solid tumor because of the immunosuppressive tumor microenvironment (TME). This affliction can be solved by vascular normalization and TME reprogramming.

**Methods:**

By using the single-cell RNA sequencing (scRNAseq) approach, we tried to find out the reprogramming mechanism that the Fc-VEGF chimeric antibody drug (Fc-VFD) enhances immune cell infiltration in the TME.

**Results:**

In this work, we showed that Fc-VEGF_121_-VEGF_165_ (Fc-VEGF chimeric antibody drug, Fc-VFD) arrests excess angiogenesis and tumor growth through vascular normalization using in vitro and in vivo studies. The results confirmed that the treatment of Fc-VFD increases immune cell infiltration including cytotoxic T, NK, and M1-macrophages cells. Indeed, Fc-VFD inhibits Lon-induced M2 macrophages polarization that induces angiogenesis. Furthermore, Fc-VFD inhibits the secretion of VEGF-A, IL-6, TGF-β, or IL-10 from endothelial, cancer cells, and M2 macrophage, which reprograms immunosuppressive TME. Importantly, Fc-VFD enhances the synergistic effect on the combination immunotherapy with anti-PD-L1 in vivo.

**Conclusions:**

In short, Fc-VFD fusion normalizes intratumor vasculature to reprogram the immunosuppressive TME and enhance cancer immunotherapy.

**Supplementary Information:**

The online version contains supplementary material available at 10.1007/s00262-022-03255-9.

## Background

The tumor microenvironment (TME) is a heterotypic pool constituted by different cell types including tumor cells, fibroblasts, stroma cells, immune cells, vasculature associated cells, and soluble factors [1]. The cell-to-cell interaction in the TME determines the property and status of cancer tissues through the secretion of cytokines and other substances throughout the environment, which is critical to tumor progression and metastasis [2, 3]. In addition, the hypoxic and oxidative stresses are the environmental stress phenotypes in the TME, which are considered the hallmarks of cancer [4]. To survive under these stresses, tumor cells activate stress-response signaling, e.g., escape from apoptosis, evasion from immune surveillance, angiogenesis, and metastasis [2, 4, 5]. As a result, many antitumor therapies targeting the members in the TME were restrained because of the immunosuppressive microenvironment [6–8]. Cancer progression in the microenvironment counts on angiogenesis to supply their need for oxygen and nutrients but also become a route to metastasis [9, 10]. Tumor angiogenesis is promoted mainly by VEGF-A secreted from tumor cells to develop new blood vessels from established vascular networks. Various cells in the TME also secrete many kinds of growth factors and cytokines, such as VEGF-A, IL-13, and IL-6 to promote angiogenesis [2, 10–12]. The imbalance between pro-angiogenic and anti-angiogenic factors leads to abnormal vasculature in the TME [13]. However, the network of tumor-associated abnormal neovessels is chaotic and leaky, which increases interstitial fluid (IFP) pressure and difficulty in material transportation. The collapsed vessels result in tumor regions that are hypoxic and acidified, which facilitates the genetic and epigenetic alterations to enhance their inflammation and aggressiveness [10]. Therefore, the highly aberrant angiogenesis contributes to maintaining the immunosuppressive TME and allows cancer cells to escape the immunosurveillance and resist immunotherapy [14]. In addition, the difficulty in vessel transportation contributes to the cancer-platelet interaction and the activation of coagulation and thromboembolism, which protects cancer cells from shear stress and immunological attack [15].

As angiogenesis is the crucial process of tumor progression, targeting angiogenesis is a desirable anti-tumor therapy. For example, targeting endothelial- and tumor-derived vascular endothelial growth factor A (VEGF-A) signaling agents have been investigated as potential cancer drugs. The anti-VEGF-A monoclonal antibody, Bevacizumab (Avastin), is the first VEGF-A targeted drug approved by the US FDA in 2004. However, VEGF-A signaling targeting has not proven as efficacious as expected because a significant fraction of patients showed the resistance to anti-angiogenic therapy. Many papers have proved that antiangiogenic therapies destroy the tumor vasculature, causing adaptive mechanisms of alternative pro-angiogenesis, activation of stem cells, and intratumoral hypoxia that will promote tumor recurrence and metastasis [8, 16–18, 18]. This reflection indicated that complete inhibition of tumor angiogenesis may not a perfect therapeutic strategy. Therefore, a strategy of vascular normalization is a solution to reduce excess angiogenesis, increase immune cells infiltration, and enhance the effect on immunotherapy [1, 14, 19–23].

In previous study, to enhance dimerization and target immune cells, we created a novel chimeric dimer fusion protein of VEGF_121_ and VEGF_165_ connected by Fc regions of human IgG1, which was called Fc-VEGF_121_-VEGF_165_ (Fc-VEGF chimeric antibody drug, Fc-VFD). The treatment of Fc-VFD, as a VEGFR2 antagonist, reduced proliferation, migration, and angiogenesis in endothelial cells and proliferation and invasion in cancer cells in vitro [9]. The aim of this study was to evaluate the efficacy of Fc-VFD on angiogenesis and tumorigenesis in vitro and in vivo and on reprogramming of TME and immune cells infiltration. In present study, we used vascular normalization as a strategy to show that the proper dose of Fc-VFD reduces excess angiogenesis and overcomes hypoxic resistance in cancer cells. Furthermore, Fc-VFD inhibits the secretion of proangiogenic factors, VEGF-A and IL-6, from cancer cells in the TME and inhibits angiogenesis and tumorigenesis. The Fc-VFD suppressed immunosuppressive TME by repressing M2 macrophages polarization and increasing immune cell infiltration using the single-cell RNA sequencing approach. Importantly, Fc-VFD enhanced vascular normalization and the synergistic effect on the combination immunotherapy with anti-PD-L1 in the mouse model. Our data demonstrated that the Fc-VFD can play a potential and critical role in the combination with cancer immunotherapy.

## Methods

### ***Purification of Fc-VEGF***_***121***_***-VEGF***_***165***_*** fusion proteins (Fc-VFD)***

Purification of Fc-VEGF_121_-VEGF_165_ fusion proteins was performed as described previously [9] and shown in the Supplementary material.

### Patients and clinical sample

Tissue specimens of 99 patients with oral squamous cell carcinoma (OSCC) were used for immunohistochemistry (IHC) analysis based on the availability of archival human tissue blocks from diagnostic resection specimens in the Departments of Pathology at Mackay Memorial Hospital, Taipei, Taiwan, with approval from the Institutional Review Board (IRB numbers 15MMHIS046 and 17MMHIS085). The main clinical characteristics of the 99 patients selected for this study are shown in a previous study [24]. All experiments were performed in accordance with relevant guidelines and regulations.

### Western blot analysis

Western blot analysis was performed as described previously [25].

### Reverse transcription-PCR (RT-PCR)

Total RNA was extracted using RNeasy® Mini Kit (74,106, QIAGEN, Germany) and reverse-transcribed using Maxima First Strand cDNA Synthesis Kit for RT-qPCR (Thermo Fisher Scientific, USA). The resulting cDNA was used as the template for PCR reactions. Real-time PCR reactions were performed by the ABI Step One Plus Real-Time PCR System (Applied Biosystems, Foster City, CA, USA) using PowerUp SYBR Green Master Mix (Applied Biosystems, Foster City, CA, USA), and the relative quantification was performed using the comparative 2-^ΔΔCT^ method. Gene-specific primers performed were described as following and in Supplementary material: Lon (5′- GTCATGGATGTTGTGGACGA-3′, 5′- GTAGTTGCGGGTGACATTGA- 3′); TGF-β1 (5′- CGACTCGCCAGAGTGGTTAT- 3′, 5′- TAGTGAACCCGTTGATGTCCA- 3′); VEGF-A (5’-AGGCCAGCACATAGGAGAGAT-3’, 5’-CTTGTCACATCTTGCAACGCGAG-3’); and primers specific to macrophages were describe in Lu et. al., 2018 [26]. All the PCR reactions were started at 94 °C for 5 min and terminated at 72 °C for 5 min. Finally, the data were analyzed using StepOne Software v2.3. Differential RNA expressions between various samples were calculated using β-actin as an internal control.

### In vivo* tumor xenograft experiment using mouse model*

BALB/C Nu and C57BL/6JNarl mice were purchased from National Laboratory Animal Center (Taiwan). The mice were aged 5 weeks and weighed 18–21 g. The HCT15 cell sediment was rinsed twice with PBS and resuspended in PBS with Matrigel Matrix (Corning), with the cell concentration regulated to 1 × 10^6^ /ml. Each mouse was injected subcutaneously at one side. After 1 week, when the tumors had grown to 50–100 mm^3^, the tumor-bearing nude mice were randomly divided into four groups (*n* = 6 per group) as follows: The control group (each mouse was intraperitoneally injected with 100 µl PBS twice a week, for a total of eight times); the Avastin group, Avastin (Roche, Basel, Switzerland) was intraperitoneally injected at different doses twice a week for a total of eight times; and the Fc-VFD group, Fc-VFD was intraperitoneally injected at different doses every other day, for a total of eight times. Starting from the first day of treatment, the tumor size was measured daily. For drugs combination experiments, B16/F10 mouse melanoma cancer cells (5 × 10^4^ cells/ml) was injected subcutaneously into C57BL/6JNar mouse followed by the same treatment of drugs *InViVo*Mab anti-mouse PD-L1 (BioXcell, SKU: BE0101), anti-mouse PD-1 (BioXcell, USA), and mouse IgG2b control (BioXcell, SKU: BE0090) by intraperitoneal injection stated above.

### Immunohistochemistry, IHC

IHC was performed as described previously [25, 27].

### Immunohistofluorescence

Paraffin-embedded sections were deparaffinized and rehydrated in graded xylene and ethanol solutions. Following antigen retrieval was performed in 10 mM (pH6.0) citrate buffer for 15 min in pressure cooker heating. Sections were blocked in 5% BSA in PBS for 30 min at room temperature in a humidified chamber. The rabbit anti-CD31 antibody (1:100, Abcam, ab28364) was applied and incubated at 4 °C for overnight. After washing, the secondary Ab was applied, and samples were further incubated for 1 h at RT. The mouse anti-αSMA antibody conjugated Alexa546 (1:50, Santa Cruz, sc-32251AF546) was applied and incubated for 1 h at room temperature. Nuclei were stained by 4',6-diamidino-2-phenylindole (DAPI). Signals were detected using a Leica TCS SP5 II confocal microscope.

### Enzyme-linked immunosorbent assay

For cell culture medium, the sample medium was centrifuged at 2000 × g for 5 min and collected the supernatants and assay according to the manufacturer’s instructions (Human IL-6 ELISA Kit, ab178013, Abcam). The blood from *orthotopic* xenograft tumor mice were collected into a serum separator tube through cardiac puncture and allowed samples to clot for 2 h at room temperature before centrifugation for 15 min at 1000 × g. Remove serum and assay immediately by ELISA kit (Mouse IL-6 ELISA Kit, ARG80199, Arigo biolaboratories, Taiwan) according to the manufacturer’s instructions. Read the OD value with a microplate reader at 450 nm and calculate the average absorbance values for each standards, controls and experimental samples.

### Preparation and analysis of for the single-cell RNA sequencing (scRNA-seq)

The details for the scRNA-seq for preparation are shown in the Supplementary material. The sample was demultiplied and barcoded using the Cell Ranger Software Suite (Version 3.1.0) (https://support.10xgenomics.com) and command cell ranger count. After obtaining the gene count for each sample, the samples were aggregated together. Eventually, the gene-barcode matrix was processed with Seurat v3 (https://satijalab.org/) and was separated with a CITE-seq-Count 1.4.3 (https://github.com/Hoohm/CITE-seq-Count). Thereafter, the following criteria were applied to each cell: the negative cells were removed. The data were normalized with a centered log-ratio (CLR) before clustering and reduction. The FindVariableFeatures function was used to scale data with the 2000 most variable genes, and the clustering and dimensionality method in R package Seurat v3. The FindAllMarkers function in R package Seurat v3 was used to perform differential analysis between the clusters. For each cluster, differentially expressed genes (DEGs) were generated relative to all other cells. FindMarkers in R package Seurat v3 was used to perform differential analysis between the pre- and post-treatments of the same cell types. Gene Ontology Biological Process (GOBP) analysis from DEGs was performed using g: Profiler (http://biit.,which also performs functional enrichment analysis known as over-representation analysis (ORA) or gene set enrichment analysis, on the input gene list. KEGG pathway analysis from DEGs was performed using Pathview (https://pathview.uncc.edu/) for identifying the clear pathway of the target cell population.

#### Statistical methods

All data were analyzed using R statistical software (version 3.1.1). Parametric Student’s t test was used to judge the significance of difference between conditions of interest. In general, a *p* value of < 0.05 was considered statistically significant (*p < 0.05, **p < 0.01, and ***p < 0.001).

## Results

### ***Fc-VEGF***_***121***_***-VEGF***_***165***_*** fusion antibody drug inhibits angiogenesis and tumor growth ***in vitro*** and ***in vivo

**Fig. 1:**
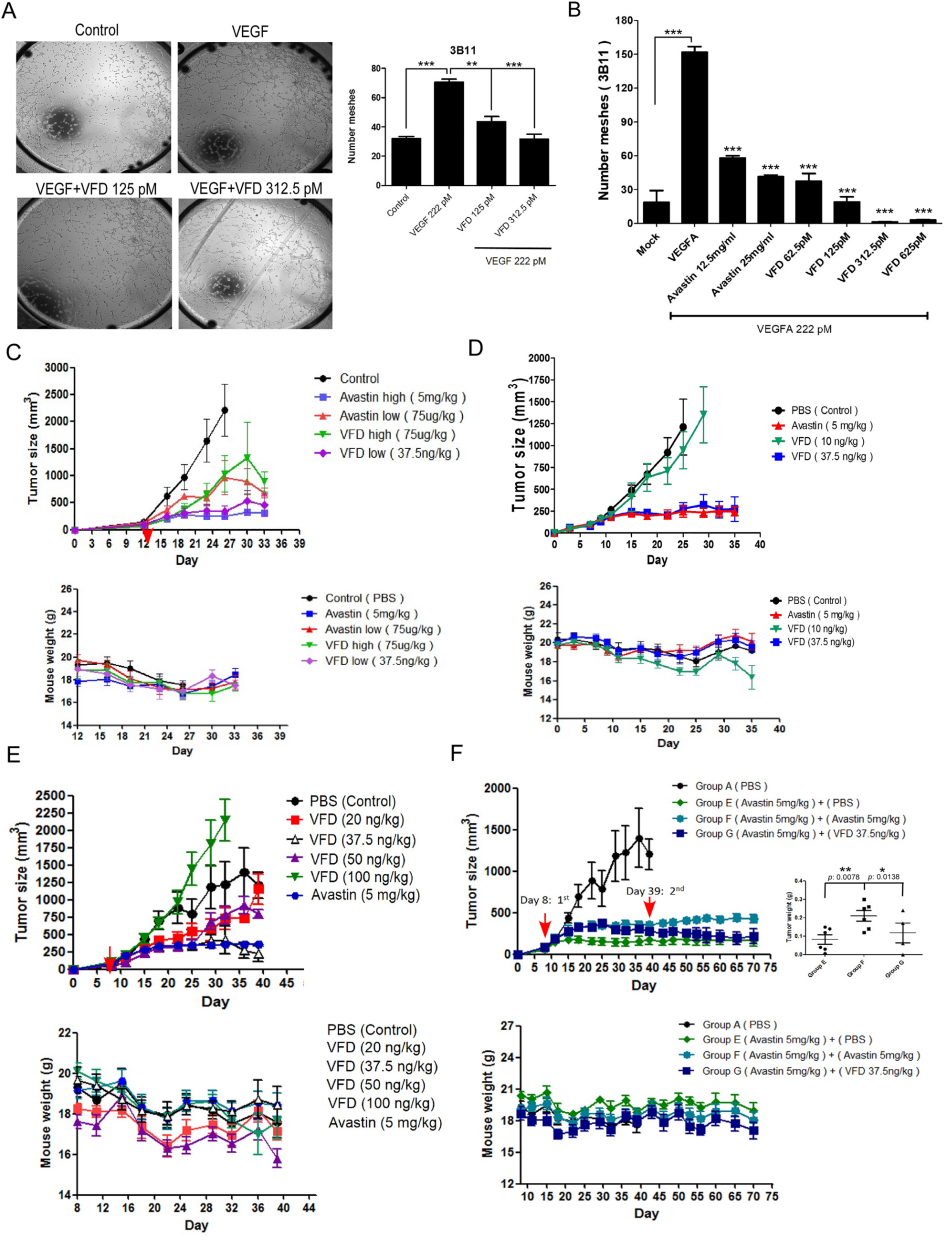
Effect of Fc-VEGF_121_-VEGF_165_ (Fc-VFD) on angiogenesis and tumorigenesis in vitro and in vivo*.* **A** 3B-11 mouse endothelial cells were inoculated on the Matrigel and treated with VEGF-A (222 pM) only or combined with Fc-VFD (125 pM or 312.5 pM). The tube formation was quantified by counting the connected cells at 200 X magnification (left panel). The error bars represent the standard deviation from at least three independent experiments (right panel). **p < 0.01; ***p < 0.001. **B** 3B-11 were treated with VEGF-A (222 pM) and combined with different concentrations of Avastin or Fc-VFD as indicated. The tube formation was quantified by counting the connected cells, and the data represented show the standard deviation from at least three independent experiments. **p < 0.01; ***p < 0.001. **C** HCT15 cells were injected subcutaneously into BALB/C Nu mice in all experiments. The mice-bearing tumor were treated with Fc-VFD (37.5 ng/kg and 75 μg/kg) or Avastin (75 μg/kg and 5 mg/kg) via intraperitoneal injection (i.p.) 12-day post-inoculation and twice a week. The tumor volume (above) and weight (below) were measured twice a week. Data represented are the mean of *n* = 6 BALB/C Nu mice. **D** The mice-bearing tumor were treated with Fc-VFD (10 ng/kg and 37.5 ng/kg) or Avastin (5 mg/kg) via intraperitoneal injection (i.p.) 12-day post-inoculation and twice a week. The tumor volume (above) and weight (below) were measured twice a week. Data represented are the mean of *n* = 6 BALB/C Nu mice. **E** The mice-bearing tumor were treated with different dosages of Fc-VFD as indicated via intraperitoneal injection (i.p.) 8-day post-inoculation and twice a week. The treatment of Avastin (5 mg/kg) was the positive control. The tumor volume (above) and weight (below) were measured twice a week. Data represented are the mean of *n* = 6 BALB/C Nu mice. **F** Fc-VFD treatment overcomes the drug-resistance of Avastin in vivo. The mice-bearing tumor were treated with Avastin (5 mg/kg) via intraperitoneal injection (i.p.) 8-day post-inoculation and twice a week for four weeks. On 39-day post-inoculation, followed by the treatment of PBS, Avastin (5 mg/kg), or Fc-VFD (37.5 ng/kg) for another four weeks. The tumor size (above) and weight (below) were measured before every injection. Data represented are the mean of *n* = 6 mice. The data were shown in the dot graphs (right panel). The error bars represent the standard deviation from six independent mice. *p < 0.05; **p < 0.01

The treatment of Fc-VFD reduced angiogenesis in endothelial cells and proliferation and invasion in cancer cells in vitro [9]. To confirm this, we performed the tube formation assay to show that the numbers of tube-like structure formation were increased by the addition of VEGF-A (Fig. 1A) and further significantly inhibited by Fc-VFD in a concentration-dependent manner under VEGF-A induction (Fig. 1A and B). We then compared the effect of Fc-VFD and Avastin (Bevacizumab, anti-VEGF-A) on angiogenesis in vitro. Compared with the control vehicle group, the Fc-VFD and Avastin at various doses significantly inhibited the tube formation (Fig. 1B). However, we found that Fc-VFD is better than Avastin at lower dosages (62.5 pM *vs.* 25 mg/ml = 127 mM) in inhibiting the tube formation in vitro. These results indicated that both Fc-VFD and Avastin significantly inhibit angiogenesis in vitro, and Fc-VFD is better than Avastin to inhibit angiogenesis in vitro at a low dosage.

We next confirmed the effect of Fc-VFD and Avastin on tumorigenesis in vivo using colorectal cancer HCT15 cells*.* Compared with the control group, we found that 75 µg/kg group of Fc-VFD and Avastin by intraperitoneal injection have a similar inhibitory effect on tumor growth (Fig. 1C); surprisingly, the low dose of 37.5 ng/kg Fc-VFD and 5 mg/kg Avastin have a similar better inhibitory effect on tumor growth (Fig. 1C). The result indicated that Fc-VFD significantly is better than Avastin at a low concentration in inhibiting tumor growth in vivo*.* To prove the dose effect of Fc-VFD, we designed a serious of in vivo experiments to test the dose range effect of Fc-VFD on tumorigenesis. Consistently, the 37.5 ng/kg group and 5 mg/kg Avastin group significantly inhibited the tumor growth in a similar efficacy, and the 20 and 50 ng/kg groups moderately inhibited the growth in mice, while the 10 ng/kg and 100 ng/kg groups almost were no significant effect on the tumor inhibition (Fig. 1D and E). These results confirmed that Fc-VFD significantly inhibits the tumor growth in vivo in a lower dosage and in a range between 20 ng/kg and 50 ng/kg. Furthermore, we found that the dose of 37.5 ng/kg Fc-VFD and dose of 5 mg/kg Avastin have a different inhibitory trend on the tumor growth when we examined carefully the two groups around Day 35 in Fig. 1C–E. The group of 5 mg/kg Avastin has a rising trend in tumor size but the trend of 37.5 ng/kg Fc-VFD group is going to decline, suggesting that anti-angiogenesis therapy using Avastin will develop drug-resistance after prolonged treatment and Fc-VFD is able to overcome the drug-resistance even prolonged treatment that will be consistent with our previous findings [9]. Since anti-angiogenesis therapy targeting VEGF-A was recently stumbled by the drug-resistance that results from adaptive mechanisms [16–18, 28], we designed a set of in vivo experiment and tried to examine the effect of Avastin and Fc-VFD on the drug resistance. We extended the treatment in mice from 4 to 8 weeks after the first-round treatment of Avastin (5 mg/kg) at Day 39 and then continue treating Avastin (5 mg/kg), Fc-VFD (37.5 ng/kg), or PBS for another 4 weeks. Consistently, the 5 mg/kg Avastin group significantly inhibited the tumor growth at the first 4 weeks compared with the control group (Fig. 1F). Most importantly, only the treatment of Fc-VFD group in the second 4 weeks inhibited a rising trend in tumor size but not in the Avastin group, showing that the Fc-VFD treatment group is able to overcome the drug-resistance after prolonged Avastin treatment (Fig. 1F). In addition, Fc-VFD treatment did not cause significant body weight loss in mice, suggesting that Fc-VFD appeared to be nontoxic to mice (Fig. 1C–F). In short, we showed that Fc-VFD significantly inhibits tumor growth at a low concentration that is better than Avastin in vivo*,* and Fc-VFD further is able to overcome the drug-resistance from prolonged Avastin treatment but not using Avastin in anti-angiogenesis therapy.

### Fc-VFD inhibits Lon-promoted tumor growth and synergizes anti-PD-L1 immunotherapy through vascular normalization

**Fig. 2:**
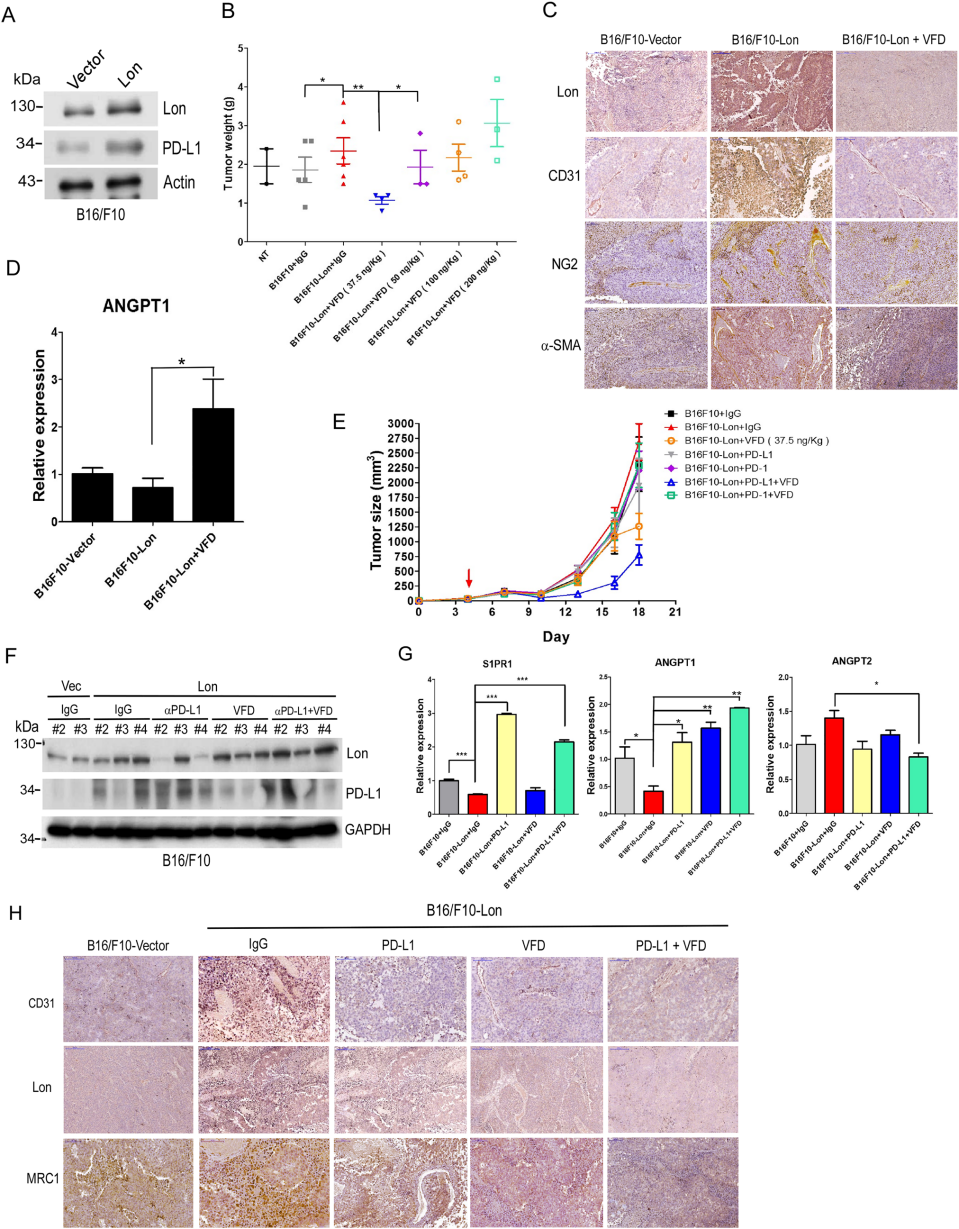
Fc-VFD inhibits Lon-induced angiogenesis and induces vessel normalization that reprograms immunosuppressive tumor microenvironment and enhances anti-PD-L1 immunotherapy in vivo. **A** Whole cell lysates from B16/F10 cells transfected with the plasmids encoding Lon or vector were analyzed by immunoblotting using the indicated antibodies. Actin as the loading control. **B** Low-dose Fc-VFD efficiently inhibits Lon-induced tumorigenesis in vivo. B16/F10 cells overexpressing Lon or not were injected subcutaneously into C57BL/6JNarl mice. The mice-bearing tumor were treated with different doses of VFD as indicated VFD via intraperitoneal injection (i.p.) 11-day post-inoculation and twice a week. The tumor weight was measured at the endpoint (25th day). NT indicated the mouse injected with B16/F10 cells only. IgG (400 μg/kg i.p.) was used in the control group. Each dot represents one mouse. *p < 0.05; **p < 0.01. **C** Fc-VFD inhibits Lon-induced tumorigenesis and induces vessel normalization in vivo. The tumor tissues were collected from the mice-bearing tumor as described in (C). Representative immunohistochemical stain of CD31, Lon, NG2, and α-SMA was performed using paraffin-embedded section of the mice tissues. Scale bar, 200 μm. **D** Fc-VFD induces vessel normalization in vivo*.* The tumor tissues were collected from the mice-bearing tumor as described in (**C**). Angiopoietin 1 (ANGPT1) expressions in tumor tissues were analyzed by qRT-PCR. The results are presented as fold increase related to the control cells (deliberately set to 1). Data are presented as mean ± SD of at least three independent experiments. The error bars shown in the graphs represent the standard deviation from at least three different experiments. *p < 0.05. **E** Fc-VFD enhances the effect of anti-PD-L1 immunotherapy on Lon-induced tumorigenesis in vivo. B16/F10 cells overexpressing Lon or not were injected subcutaneously into C57BL/6JNarl mice (*n* = 6). The mice-bearing tumor were treated with either anti-PD-L1/PD-1 antibody (8 mg/kg), VFD (37.5 ng/kg), or the combination of anti-PD-L1 and VFD as indicated via intraperitoneal injection (i.p.) 4-day post-inoculation and twice a week. The tumor weight was measured at the endpoint (18th day). IgG (400 μg/kg i.p.) was used in the control group. Each dot represents one mouse. **F** The tumor tissues were collected from the mice-bearing tumor as described in (**B**). The protein expression levels were analyzed by western blot using the indicated antibodies. GAPDH as the loading control. **G** The combination of Fc-VFD and anti-PD-L1 induces vessel normalization in vivo. The tumor tissues were collected from the mice-bearing tumor as described in (**B**). The expression of S1PR1, Angiopoietin 1 (ANGPT1), and ANGPT2 in tumor tissues was analyzed by qRT-PCR. The results are presented as fold increase related to the control cells (deliberately set to 1). Data are presented as mean ± SD of at least three independent experiments. The error bars shown in the graphs represent the standard deviation from at least three different experiments. *p < 0.05; **p < 0.01; ***p < 0.001. **H** The combination of Fc-VFD and anti-PD-L1 induces vessel normalization in vivo. The tumor tissues were collected from the mice-bearing tumor as described in (**B**). Representative immunohistochemical stain of CD3, Lon, and MRC1 was performed using paraffin-embedded section of the mice tissues. Scale bar, 200 μm

**Fig. 3:**
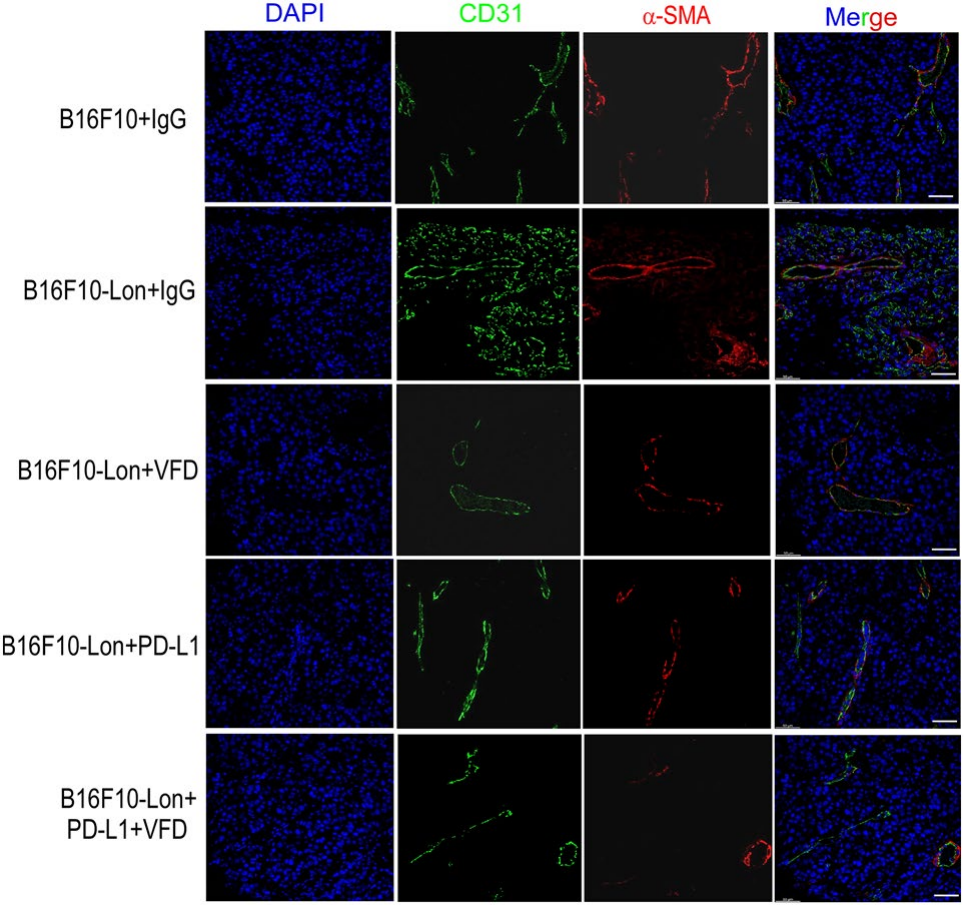
Fc-VFD induces vessel normalization and enhances anti-PD-L1 immunotherapy in vivo. The tumor tissues were collected from the mice-bearing tumor as described in Fig. 2E. Representative immunohistofluorescence stains of CD31 and α-SMA were performed using paraffin-embedded section of the mice tissues. Scale bar, 50 μm. CD31 in green and α-SMA in red

Immunotherapy is an emerging cancer therapy with potential great success; however, one type of cancer immunotherapy, immune checkpoint inhibitor (anti-PD-1/PD-L1), has response rates of only 10–30% in the clinical treatment [29–33] because of immunosuppressive TME [10]. Thus, we tried to examine whether Fc-VFD improves the immunosuppressive TME by vascular normalization to enhance the effect of anti-PD-L1 immunotherapy on tumor inhibition. We previously found that mitochondrial Lon induces ROS-dependent production of inflammatory cytokines, IL-6, IL-13, and VEGF-A, that consequently induce angiogenesis, M2 macrophage polarization, and immunosuppressive TME [2]. Since mitochondrial Lon also induces ROS-dependent induction of PD-L1 [3], we verified that PD-L1 was induced under Lon upregulation in B16/F10 cells both in mRNA and protein level (Figs. 2A and 5A). We next performed animal experiments to examine the dose effect of Fc-VFD on tumorigenesis. We found that Lon upregulation in B16/F10 melanoma (Fig. 2B) and OEC-M1 oral cancer (Fig. S1) induced tumor growth. Fc-VFD of 37.5 ng/kg group significantly inhibited the tumor growth by intraperitoneal injection; the 50 ng/kg group moderately inhibited the growth, while the 100 ng/kg and 200 ng/kg groups almost were no significant effect on the tumor inhibition in mice (Fig. 2B). Furthermore, the range of effective Fc-VFD dosages was further validated using intravenous injection on the OECM-1 tumor xenograft model. Consistently, the 75 ng/kg group by intravenous injection showed the most valuable inhibition on the Lon-induced tumorigenesis (Fig. S1). To examine whether that the treatment of Fc-VFD represses excess tumor angiogenesis and promotes vascular normalization, we first monitored the expression level of CD31, an endothelial cell marker, by immunohistochemical stain. The results showed that CD31 expression was dramatically increased in B16/F10 Lon-overexpressing tumor and decreased under Fc-VFD treatment (Fig. 2C), suggesting that Fc-VFD treatment is able to inhibit excess angiogenesis and promote vascular normalization. Similar results were observed in the expression level of NG2 (a pericyte marker) and α-SMA, and Fc-VFD treatment reversed the level of NG2 and α-SMA in xenograft B16/F10 Lon-overexpressing tumors (Fig. 2C). We further confirmed that Lon upregulation induced the decrease in Angiopoitetin 1 (ANGPT1), a vessel maturation-related protein, and Fc-VFD treatment reversed the expression of ANGPT1 in xenograft B16/F10 Lon-overexpressing tumors (Fig. 2D). We next injected Lon-overexpressing B16/F10 mouse melanoma cells in C57BL/6JNarl mice treated with Fc-VFD, anti-PD-1, or anti-PD-L1 (400 μg/kg) alone or combination. The results showed that Lon-overexpressing tumors grew with control treatment, but the tumor size was decreased when treated with Fc-VFD or anti-PD-L1 (Fig. 2E). Moreover, the combination of Fc-VFD with anti-PD-L1 showed significantly better inhibitory effects on tumor growth in mice compared with single treatment (Fig. 2E). In addition, the combination of Fc-VFD and anti-PD-L1 did not cause significant weight loss, which means that the combination with appropriate dosages is non-toxic to mice (data not shown). Western results also showed that PD-L1 expression was decreased under Fc-VFD treatment compared to the IgG control (Fig. 2F). We further confirmed that Fc-VFD treatment induces vascular normalization by monitoring vessel maturation-related protein, ANGPT1, and pro-angiogenic protein, ANGPT2 in vivo*.* The results indicated that ANGPT1 was downregulated in Lon-overexpressing B16/F10 group and significantly upregulated after treatment with Fc-VFD and/or anti-PD-L1; ANGPT2 had an opposite trend to ANGPT1 in orthotopic-xenograft tumors (Fig. 2G). Immunohistochemistry (IHC) stain of CD31, an endothelial cell marker, also showed that CD31 expression was dramatically increased in Lon-overexpressing B16/F10 tumor and decreased under the treatment with Fc-VFD and/or anti-PD-L1 (Fig. 2H), suggesting that Fc-VFD treatment is able to promote vascular normalization. The findings of vascular normalization were further confirmed by the immunohistofluorescence experiment. The results showed that the expression CD31 and α-SMA was intensely increased in the Lon-overexpressing tumor and decreased under the treatment with Fc-VFD and/or anti-PD-L1 (Fig. 3). To find the clinical significance of Lon-induced angiogenesis in cancer progression, we examined whether the CD31 marker regulated by Lon is clinically relevant in oral cancer, OSCC. The expression pattern of Lon and CD31 in 99 samples of tumor tissues from OSCC patients was determined by IHC analysis. The main clinicopathological characteristics of the patients in this study are as described in our previous report [24]. The association between Lon and CD31 expression in OSCC tissues was tested in the contingency table using Fisher’s exact test. The result showed that CD31 expression showed a significant correlation with Lon expression (*P* = 0.000497, Table 1). Consistently, the correlation between Lon and CD31 expression is statistically significant by Spearman’s rank test (*P* = 0.00162, Table 1).

**Table 1:** Contingency table shows a positive association between Lon and CD31, based on 99 OSCC patients with Lon/CD31 protein staining

		*Lon*				Fisher, *P*	Spearman's
		None	Weak	Median	Strong		Rank Correlation (ρ, *P*)
*CD31*	None	11	5	9	16	0.000497	0.241, 0.0162
	Weak	0	6	7	1		
	Median	2	3	7	11		
	Strong	2	3	1	15		

### Fc-VFD inhibits Lon-induced angiogenesis through immune cell infiltration and M2 macrophages polarization

**Fig. 4:**
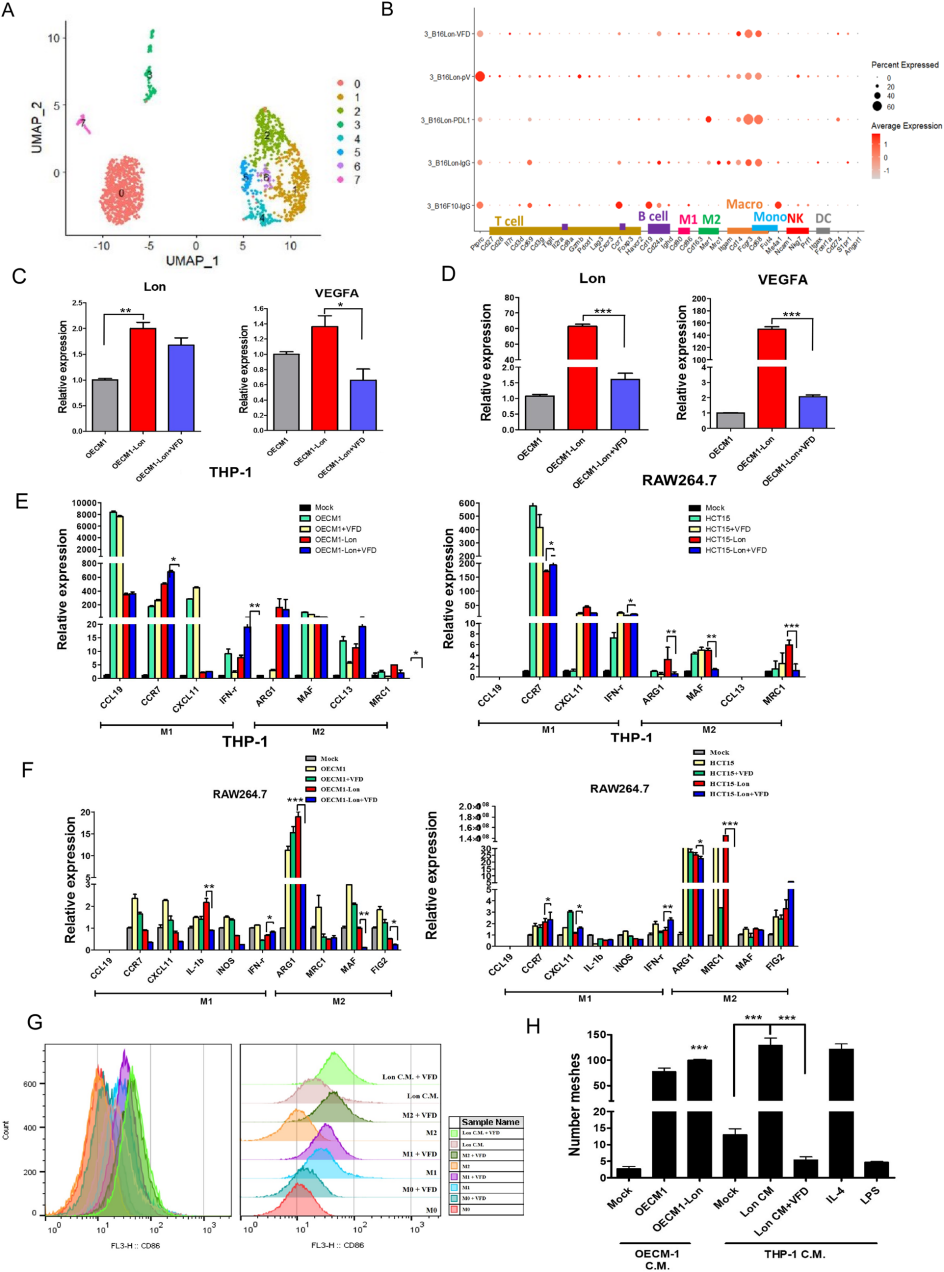
Fc-VFD induces immune cell infiltration and inhibits Lon-induced M2 macrophage polarization and angiogenesis in the tumor microenvironment. **A** and **B** Fc-VFD reprograms immunosuppressive microenvironment and enhances immune cell infiltration in tumor in vivo shown by the single-cell RNA sequencing (scRNA-seq) approach*.* Integrated cluster map of B16/F10 mouse melanoma subcutaneous injected tumors. UMAP plot of five groups with different treatment B16/F10 mouse melanoma cells subcutaneous injection tumors. UMAP plots are reveling eight different clusters (**A**). The tumor tissues were collected from the mice-bearing tumor as described in (Fig. 2B). Cluster 3 was found from the UMAP plots of the tumor tissues by the 10X Genomics scRNA-seq method. Analysis of 10X Genomics scRNA-seq confirmed an increase in killing immune cells including cytotoxic T, M1-macrophages, and NK cells. VFD represents Fc-VFD (37.5 ng/kg) only; PD-L1 represents anti-PD-L1 antibody (400 μg/kg) only; pV represents the combination of anti-PD-L1 and VFD. The percentages of gene expression are denoted by the size of spot. The brightness of red color is denoted average of expression (**B**). **C** and **D** Fc-VFD inhibits Lon-induced VEGF-A by cancer in macrophage. The conditioned medium from OECM-1 cells transfected with Lon plasmid and treated with Fc-VFD (312.5 pM) or not for 24 h were collected. THP-1 monocytes treated with PMA (Phorbol 12-myristate 13-acetate) 100 ng/ml for 6 h (**C**) and RAW264.7 macrophage (**D**) were incubated with the conditioned medium. The mRNA expression of Lon and VEGF-A in macrophages was analyzed by quantitative real-time PCR. The results are presented as fold increase related to the control cells (deliberately set to 1). Data are presented as mean ± SD of at least three independent experiments. The error bars shown in the graphs represent the standard deviation from at least three different experiments. *p < 0.05; **p < 0.01, ***p < 0.001. **E** and **F** Fc-VFD inhibits Lon-induced M2 macrophage polarization by cancer. The conditioned medium from OECM-1 or HCT15 cells transfected with Lon plasmid and treated with Fc-VFD (312.5 pM) or not for 24 h were collected. THP-1 monocytes treated with PMA (Phorbol 12-myristate 13-acetate) 100 ng/ml for 6 h (**E**) and RAW264.7 macrophages (**F**) were incubated with the conditioned medium as indicated. The mRNA expression of macrophage-specific marker genes was analyzed by quantitative real-time PCR as indicated. The results are presented as fold increase related to the control cells (deliberately set to 1). Data are presented as mean ± SD of at least three independent experiments. The error bars shown in the graphs represent the standard deviation from at least three different experiments. *p < 0.05; **p < 0.01, ***p < 0.001. **G** Fc-VFD repolarizes Lon-induced M2 macrophage toward to M1 type. The conditioned medium (C.M.) from OECM-1 cells transfected with Lon plasmid for 48 h were collected. THP-1 monocytes treated with PMA (Phorbol 12-myristate 13-acetate) 100 ng/ml for 24 h and reseed 1 × 10^6^ cells for another 16 h. THP-1 macrophage was polarized to M1 by LPS (100 ng/ml) and INF-γ (20 ng/ml) and M2 by IL-4 (20 ng/ml) and IL-13 (20 ng/ml), respectively. THP-1 cells were incubated with C.M. as indicated and treated with Fc-VFD (312.5 pM) for 24 h. The M1 macrophage population were examined by flow cytometry with M1 macrophage marker, CD86. **H** Fc-VFD inhibits M2 macrophage-induced angiogenesis by Lon in the tumor microenvironment. The conditioned medium (C.M.) from OECM-1 transfected with Lon plasmid or not were collected. THP-1 cells were incubated with the C.M. from OECM-1 and treated with Fc-VFD (312.5 pM) or not for 24 h and the C.M. from THP-1 cells were collected (THP-1 C.M.). 3B-11 cells on the Matrigel were treated with the THP-1 C.M. The tube formation was quantified by counting the connected cells. THP-1 macrophage was polarized to M1 and M2 macrophages by LPS (100 ng/ml) and IL-4 (20 ng/ml), respectively, which was used both as the positive and negative control. The data were shown in the bar graphs. The error bars represent the standard deviation from at least three independent experiments. ***p < 0.001

We next verified that Fc-VFD enhances the effect of anti-PDL1 immunotherapy by immune cell infiltration and polarization in the TME. We analyzed gene expression profiling of the Lon-overexpressing B16/F10 tumor under the treatments using the single-cell RNA sequencing. The results showed that the cluster 3 showed an increased expression of Ptprc (CD45) that is defined as immune cells (Fig. 4A). The results indicated that gene expression of cytotoxic T-cells, CD8 and Gzmb, and other T-cells, CD27 and CD28, were higher in the tumor treated with combination of anti-PD-L1 and Fc-VFD than treated with the single treatment. Moreover, gene expression of M1-type macrophages (CD80/86) and NK cells (NKG7, PRF1) were also higher in the combination treatment (Fig. 4B). These results showed that Fc-VFD prevents excess tumor angiogenesis and facilitates immune cell infiltration via vascular normalization to enhance cancer immunotherapy by anti-PDL1. As we found that Fc-VFD inhibits angiogenesis and facilitates immune cell infiltration in the TME, we confirmed that Fc-VFD would affect macrophages polarization in the TME. Previous studies showed that Lon-induced inflammatory cytokines activate Lon and VEGF-A expression in macrophages in a paracrine and autocrine manner [2]. Indeed, the real-time quantitative PCR results showed that Lon and VEGF-A were induced in THP-1 (Fig. 4C) and RAW264.7 (Fig. 4D) macrophage cells under the treatment with media from Lon-overexpressing cancer cells, and Fc-VFD showed repressive effects on the induction in VEGF-A expression in macrophages (Fig. 4C and D). Previously, we found that Lon in cancer cells induces p38- and NF-κB-dependent inflammatory response to promote M2 macrophages polarization [2]. In macrophage cells, Lon-induced M2 macrophage markers, such as ARG1, MAF, CCL13, and MRC1 were repressed by treatment with Fc-VFD, and Lon-inhibited M1 macrophage marker, CD86, was increased by treatment with Fc-VFD (Fig. 4E and F). These results indicated that Fc-VFD represses inflammatory cytokines secreted from Lon-overexpressed cancer cells and M2 macrophage polarization in an autocrine manner. Similar results also were found that M2 marker MRC1 expression was dramatically increased in Lon-overexpressing B16/F10 tumor and decreased under the treatment with Fc-VFD and/or anti-PD-L1 (Fig. 2H), and Fc-VFD induced the M1-type of THP-1 and the treatment with the M2 inducer and the Lon-overexpressing cancer C.M. as negative controls (Fig. 4G). To further confirm that Lon-induced VEGF-A expression in M2 macrophages promotes angiogenesis, we used the media from Lon-overexpressing cancer cells and from THP-1 treated with the above media to treat 3B-11 endothelial cells. These data showed that angiogenesis of 3B-11 cells were increased after treatment with the media from the induced M2 macrophages using tube formation assay (Fig. 4H). The data further indicated that VFD significantly inhibits M2 macrophage-induced angiogenesis (Fig. 4H). Together, these results showed that Fc-VFD enhances cancer immunotherapy by preventing excess tumor angiogenesis of endothelial cells and infiltration of M2 macrophage in the TME.

### Fc-VFD inhibits Lon-induced angiogenic cytokine released from cancer cells in the tumor microenvironment

**Fig. 5:**
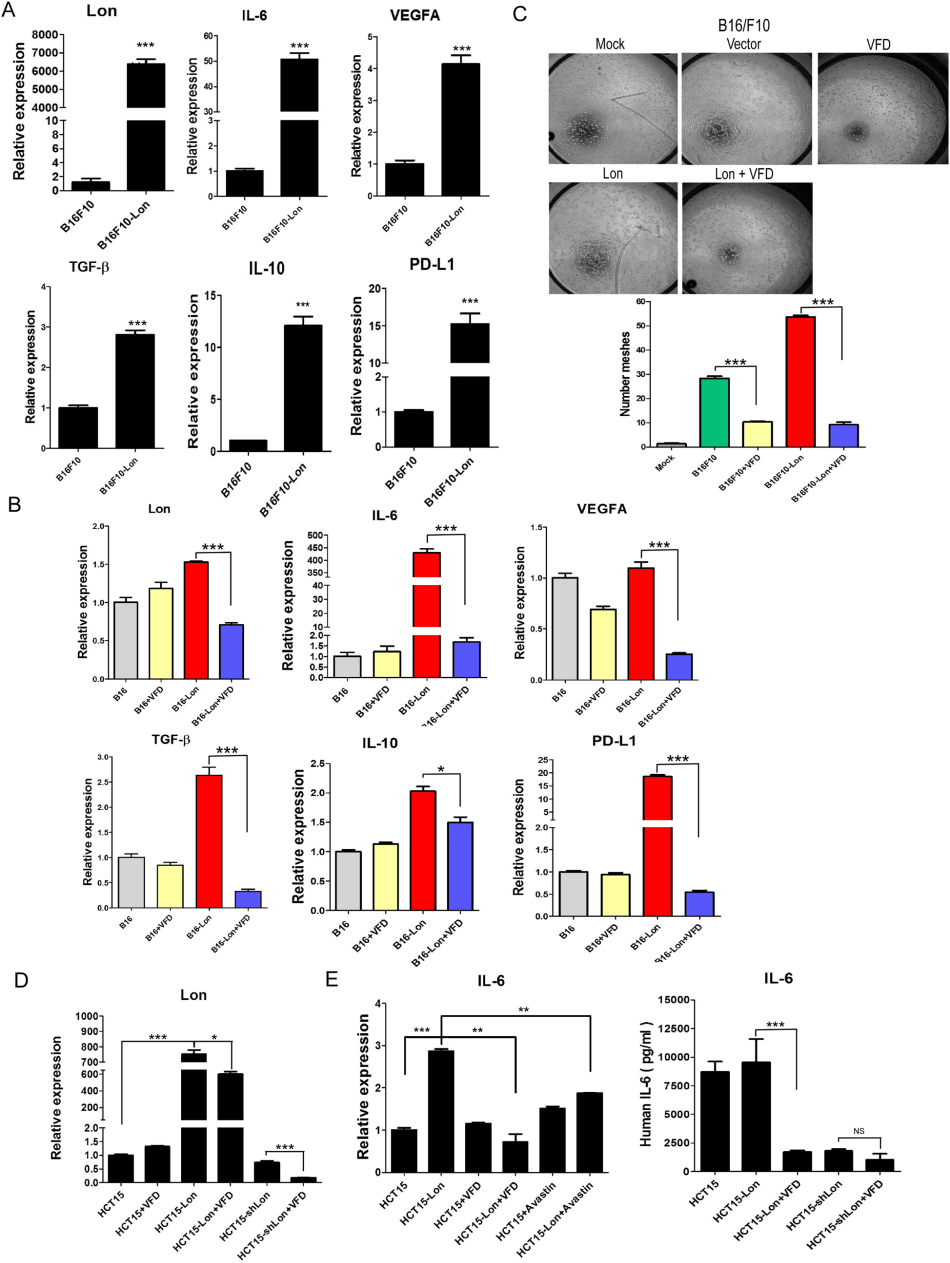
Fc-VFD inhibits Lon-induced expression of inflammatory cytokines and angiogenesis in cancer. **A** B16/F10 mouse melanoma cells were transfected with the plasmid encoding Lon. The mRNA expression of Lon, IL-6, VEGF-A, TGF-β, IL-10, and PD-L1 was analyzed by quantitative real-time PCR. The results are presented as fold increase related to the control cells (deliberately set to 1). Data are presented as mean ± SD of at least three independent experiments. The error bars shown in the graphs represent the standard deviation from at least three different experiments. *p < 0.05; ***p < 0.001. **B** B16/F10 cells were transfected with Lon plasmid and treated with Fc-VFD (312.5 pM) for 24 h or not. The mRNA expression was analyzed by quantitative real-time PCR. The results are presented as fold increase related to the control cells (deliberately set to 1). Data are presented as mean ± SD of at least three independent experiments. The error bars shown in the graphs represent the standard deviation from at least three different experiments. *p < 0.05; ***p < 0.001. **C** Conditioned medium was collected from B16/F10 cells transfected with Lon plasmid or not and treated with Fc-VFD (312.5 pM) or not for 24 h. 3B-11 cells were treated with the conditioned medium. The tube formation was quantified by counting the connected cells at 200 X magnification (top panel). The data were shown in the bar graphs (bottom panel). The error bars represent the standard deviation from at least three independent experiments. ***p < 0.001. **D** HCT15 cells were transfected with the plasmids encoding Lon or Lon-shRNA and treated with or without Fc-VFD for 24 h. The mRNA expression of Lon was analyzed by quantitative real-time PCR. *p < 0.05; ***p < 0.001. **E** HCT15 cells were transfected with the plasmids encoding Lon or Lon-shRNA and treated with or without Fc-VFD for 24 h. The mRNA expression of IL-6 was analyzed by quantitative real-time PCR (left). IL-6 secretion from the treated cells was analyzed by ELISA (right). The data were shown in the bar graphs. The error bars represent the standard deviation from at least three independent experiments. *p < 0.05; ***p < 0.001

We tried to find out the mechanism that Fc-VFD enhances immune cell infiltration in the TME. Since Fc-VFD has been showed to inhibit autocrine VEGFR2-HIF-1α/Lon signaling [9], we then proved whether Fc-VFD can repress Lon-induced inflammatory cytokines from cancer cells. Lon upregulation induced the induction of inflammatory cytokines and their downstream signaling, such as IL-6, VEGF-A, TGF-β, INF-γ, and PD-L1 (Fig. 5A) and Fc-VFD also inhibited the Lon-induced expression of these cytokines from B16/F10 cells (Fig. 5B) and angiogenesis (Fig. 5C). In order to confirm Lon-induced inflammatory cytokines were not only found in a single type of cancer, but also we overexpressed Lon in HCT15 colorectal cancer cells. Fc-VFD indeed was able to inhibit the level in Lon expression in HCT15 cells (Fig. 5D). We showed that Lon overexpression-induced production of IL-6 was inhibited by Fc-VFD in the RNA and protein level in HCT15 cells (Fig. 5E). Furthermore, Lon overexpression-induced angiogenesis was inhibited by Fc-VFD (Fig. S2). Together, Fc-VFD not only targets angiogenesis of endothelial cells, but also inhibits angiogenic cytokine signaling from Lon-overexpressed cancer cells in the TME.

### Fc-VFD inhibits angiogenesis of endothelial cells by repressing VEGF-A and IL-6 signaling in the tumor microenvironment

**Fig. 6:**
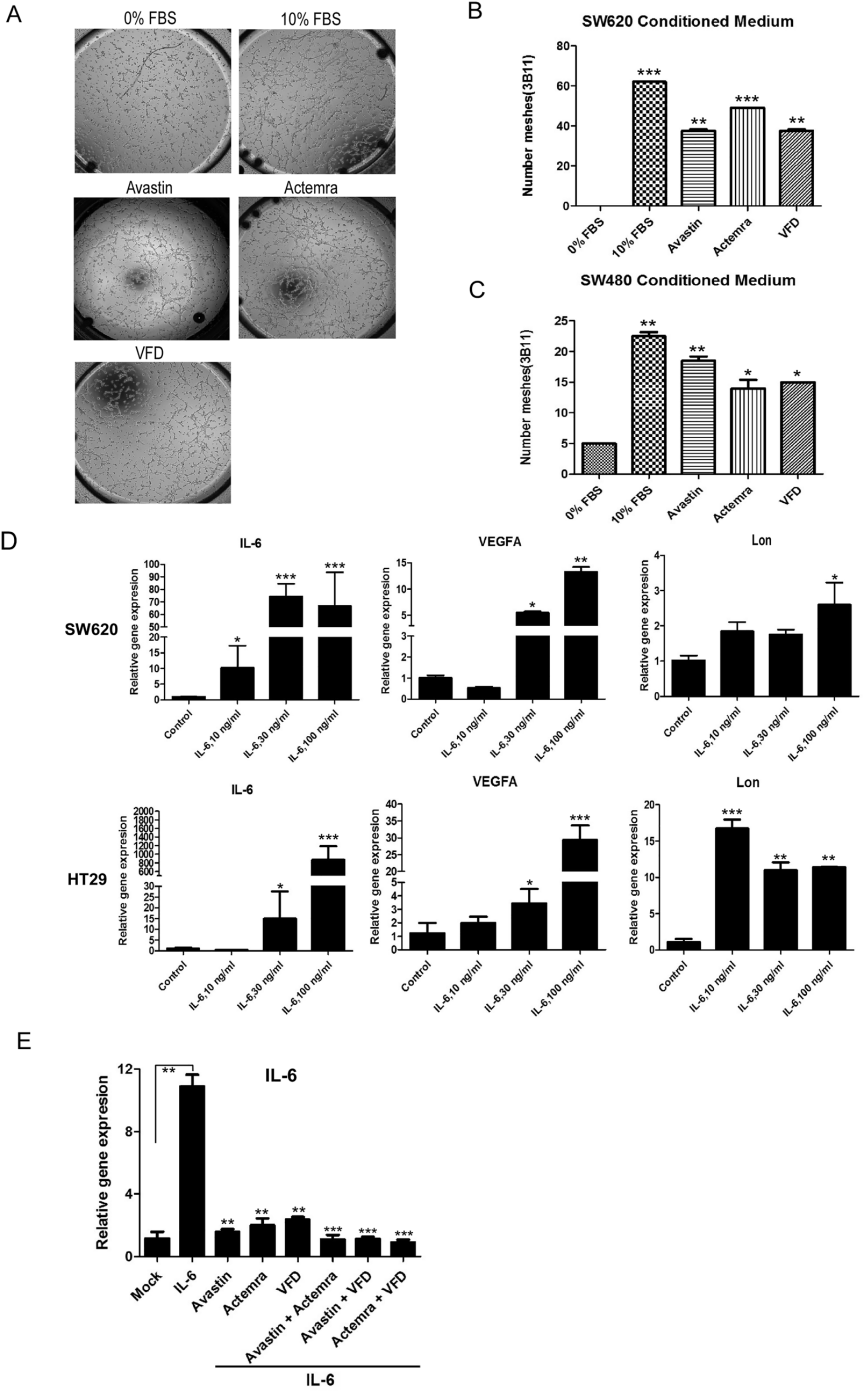
Fc-VFD inhibits angiogenesis induced by VEGF-A and IL-6 from cancer cells. **A**–**C** Fc-VFD inhibits the tube formation that induced by VEGF-A and IL-6. Conditioned medium from SW620 or SW480 cells were incubated with Avastin (25 μg/ml), Actemra (20 μg/ml), or Fc-VFD (312.5 pM) for 24 h. 3B-11 cells were treated with the conditioned medium. The tube formation was quantified by counting the connected cells (**A**). The data with SW620 (**B**) or SW480 (**C**) conditioned medium were shown in the bar graphs. The error bars represent the standard deviation from at least three independent experiments. *p < 0.05; **p < 0.01; ***p < 0.001. **D** IL-6 induces the expression of VEGF-A and Lon SW620 and HT29 cancer cells. SW620 and HT29 cells were treated with different doses of IL-6 as indicated for 24 h. The mRNA expression of IL-6, VEGF-A, and Lon were analyzed by quantitative real-time PCR. The results are presented as fold increase related to untreated cells (deliberately set to 1). Data are presented as mean ± SD of at least three independent experiments. The error bars shown in the graphs represent the standard deviation from at least three different experiments. *p < 0.05; **p < 0.01; ***p < 0.001. **E** HT29 were treated with IL-6 (30 ng/ml) only or combined with Avastin (25 μg/ml), Actemra (20 μg/ml), or VFD (312.5 pM) for 24 h. IL-6 mRNA expression was analyzed by quantitative real-time PCR

**Fig. 7:**
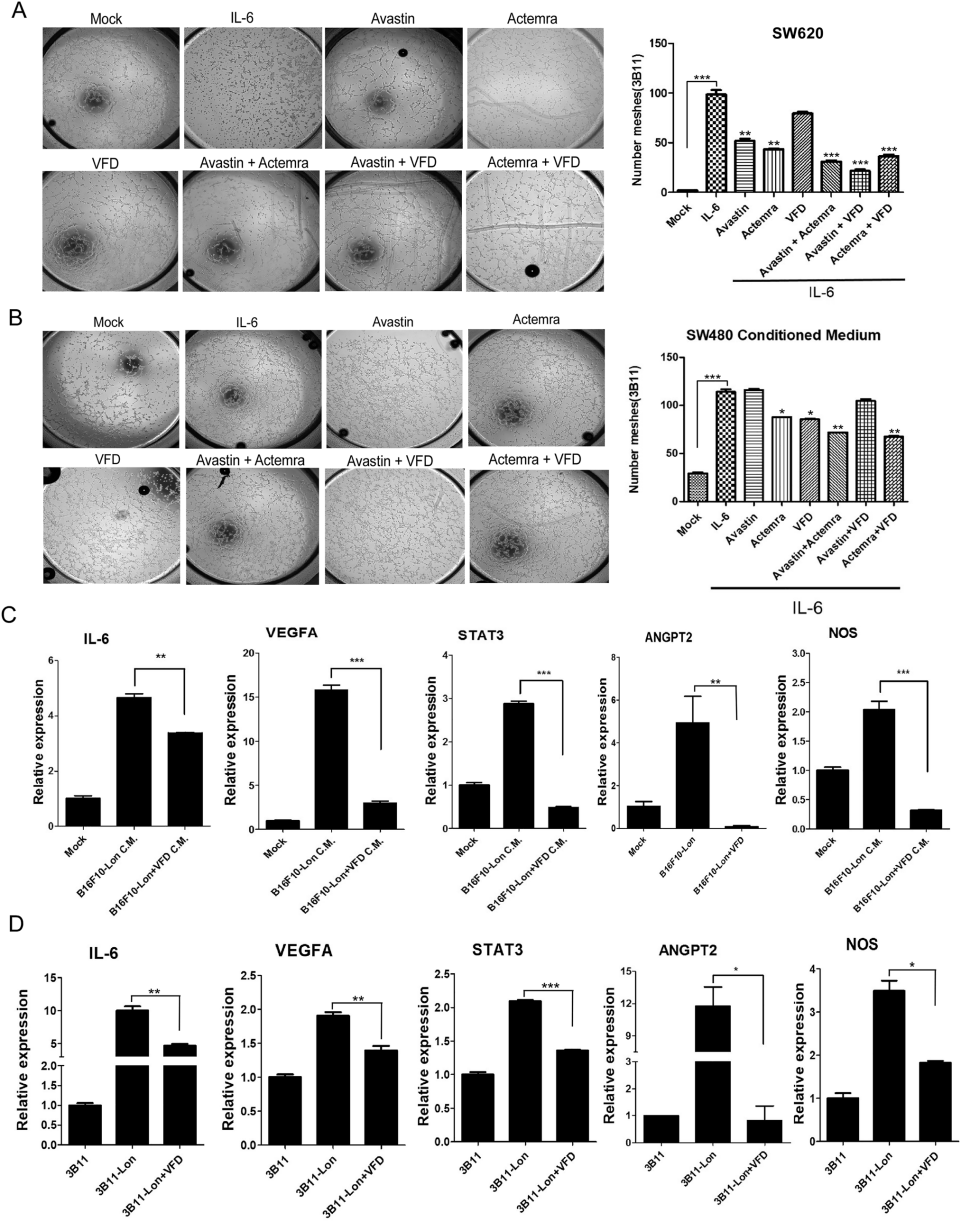
Fc-VFD inhibits Lon-induced angiogenesis and induces vessel normalization in endothelial cells in vitro. **A** Conditioned medium from SW620 cells incubated with IL-6 (30 ng/ml) only or combined with Avastin (25 μg/ml), Actemra (20 μg/ml), Fc-VFD (312.5 pM), or their combination as indicated for 24 h. 3B-11 cells were treated with the conditioned medium. The tube formation was quantified by counting the connected cells (left panel). The data were shown in the bar graphs (right panel). The error bars represent the standard deviation from at least three independent experiments. **p < 0.01; ***p < 0.001. **B** SW480 conditioned medium induced angiogenesis was blocked by Fc-VFD. Conditioned medium from SW480 cells were treated with IL-6 (30 ng/ml) or combined with Avastin (25 μg/ml), Actemra (20 μg/ml), or Fc-VFD (312.5 μM). 3B-11 cells were treated with the indicated CM. The tube formation was quantified by counting the connected cells. The panels represent the standard deviation from at least three independent experiments. **p < 0.01; ***p < 0.001. **C** Fc-VFD inhibits Lon-induced pro-angiogenic factors by cancer in 3B-11 endothelial cells. The conditioned medium from B16/F10 cells transfected with Lon plasmid and treated with Fc-VFD (312.5 pM) or not for 24 h were collected. 3B-11 mouse endothelial cells were incubated with the conditioned medium. The mRNA expression in 3B-11 was analyzed by quantitative real-time PCR as indicated. The results are presented as fold increase related to the control cells (deliberately set to 1). Data are presented as mean ± SD of at least three independent experiments. The error bars shown in the graphs represent the standard deviation from at least three different experiments. *p < 0.05; **p < 0.01, ***p < 0.001. **D** Fc-VFD inhibits Lon-induced pro-angiogenic factors in 3B-11 endothelial cells. 3B-11 cells were transfected with the plasmids encoding Lon and treated with Fc-VFD (312.5 pM) or not for 24 h. The mRNA expression in 3B-11 was analyzed by quantitative real-time PCR as indicated. The results are presented as fold increase related to the control cells (deliberately set to 1). Data are presented as mean ± SD of at least three independent experiments. The error bars shown in the graphs represent the standard deviation from at least three different experiments. *p < 0.05; **p < 0.01, ***p < 0.001

Our recent finding that mitochondrial Lon induces NF-κB-dependent IL-6 and VEGF-A secretion to promote angiogenesis [2]. In SW620, higher expression level of Lon along with higher VEGF-A expression than that in SW480 was observed (Fig. S3), implying that the secretion of VEGF-A and IL-6 from cancer cells is Lon-dependent. 3B-11 cells were cultured in the conditioned media from SW480 and SW620 treated with Avastin, Actemra (tocilizumab, anti-IL6R), or Fc-VFD and a tube network assay was performed. The conditioned media from colorectal cancer cells increase numbers of tube formation. The blockade of VEGF-A and IL-6 signaling by Avastin, Fc-VFD, and Actemra inhibits cancer-induced angiogenesis of endothelial cells (Fig. 6A–C). IL-6 is a pleiotropic cytokine and has been proved to mediate angiogenesis in the TME [2, 34–36]. Since it is necessary to understand how Fc-VFD targets cancer cells, we showed that the IL-6 and VEGF-A expression in both SW620 and HT29 cells follows a dose-dependent manner (Fig. 6D). The increasing IL-6 expression by additional IL-6 was repressed by VEGF-A and IL-6 inhibitors and even stronger inhibition under Fc-VFD combination treatments (Fig. 6E). These results showed that Fc-VFD has inhibition ability on cancer cells through inhibiting VEGF-A and IL-6 signaling and on angiogenesis of endothelial cells similar to the effect of Avastin and Actemra. In addition, the tube formation induced by IL-6 was inhibited by these inhibitors and better inhibition effects with combination treatments (Fig. 7A and B). To further verify that Fc-VFD inhibits Lon-induced angiogenesis of endothelial cells, we overexpressed Lon in B16/F10 mouse melanoma cells and collected the conditioned medium (CM) from the melanoma cells to treat endothelial cell 3B-11. The results showed that the treatment causes an induction of IL-6, STAT3, VEGF-A, nitric oxide synthase (NOS), and angiopoietin 2 (ANGPT2); however, Fc-VFD can reverse the trend in 3B-11 cells under the treatment of CM from the B16/F10 cells (Fig. 7C). On the other hand, the Fc-VFD treatment increased the level of ANGPT1, PDGFB, and S1PR1, a vessel differentiation protein in endothelial cells (Fig. S4). Similarly, Lon upregulation in 3B-11 cells causes an induction of IL-6, STAT3, VEGF-A, NOS, and ANGPT2 in the cells (Fig. 7D; Fig. S5), and Fc-VFD treatment inhibited their expression and induced the expression of vessel differentiation proteins in 3B-11 cells when Lon was upregulated in the cells (Fig. 7D; Fig. S4). Taken together, these findings conclude that Fc-VFD inhibits the angiogenesis of endothelial cells by the inhibition of VEGF-A and IL-6 expression both from cancer and endothelial cells in the TME.

## Discussion

**Fig. 8:**
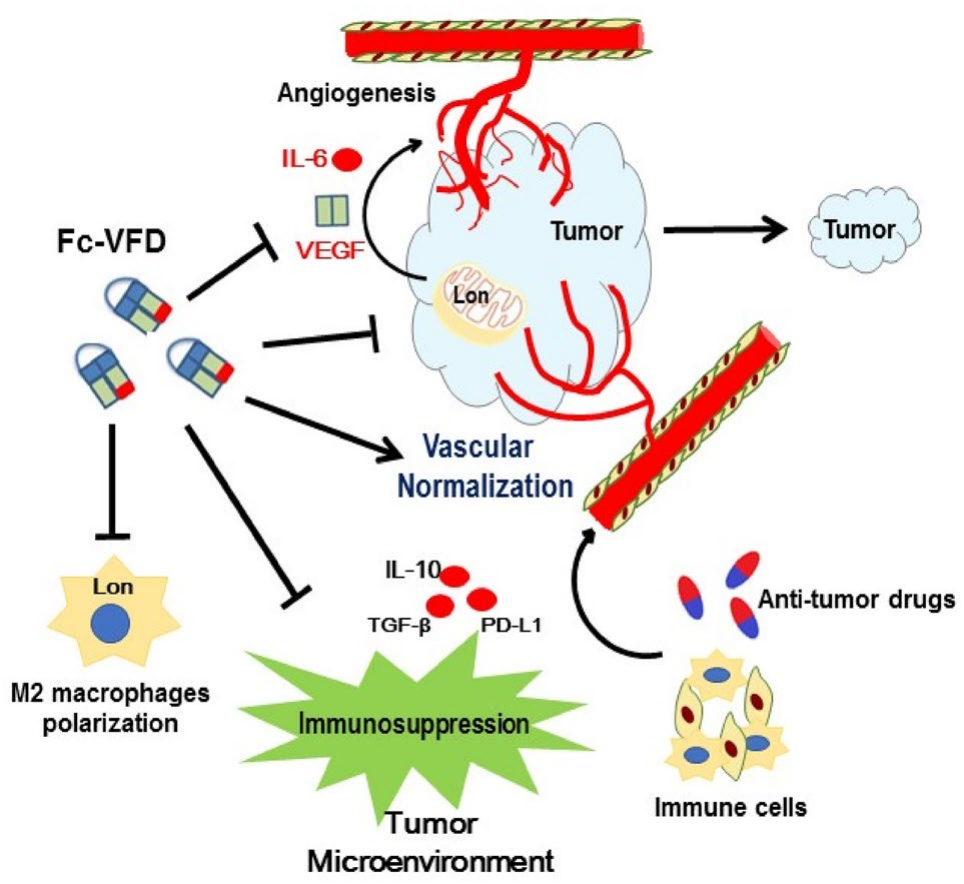
Scheme of Fc-VEGF_121_-VEGF_165_ (Fc-VFD) inhibits Lon-induced angiogenesis and tumorigenesis by targeting VEGF-A and IL-6 and enhances immunotherapy via vessel normalization and reprogramming immunosuppressive tumor microenvironment

In this study, we have shown that a chimeric dimer fusion protein, Fc-VFD [9] can arrest the angiogenesis of endothelial cells and interfere with the growth and hypoxic resistance of cancer cells in vitro and in vivo. Furthermore, the Fc-VFD inhibits the secretion of proangiogenic factors, VEGF-A and IL-6, from cancer and macrophage cells in the tumor microenvironment (TME). We further found that Fc-VFD inhibits tumorigenesis through vascular normalization and reprogramming of TME that increases immune cells infiltration and represses M2 macrophages polarization. Importantly, Fc-VFD enhances the synergistic effect on the combination therapy with anti-PD-L1 in vivo mouse model (Fig. 8).

In this study, we showed that Fc-VFD contributes to the reprogramming of the TME via vascular normalization that increases immune cells infiltration and represses M2 macrophages polarization. A strategy of vascular normalization was used to increase immune cells infiltration that further enhances immunotherapy [14, 20, 22, 23]. In the past decade, there are enormous and revolutionary progressions happen in the area of cancer treatment, that is the rise of cancer immunotherapy [37]. However, cancer immunotherapy of immune checkpoint inhibitors alone showed a low response rate in solid tumors [38]. The combination of other therapies with immunotherapy recently showed an increasing cure rate of cancer [39, 40]. On the other hand, the efficacy of cancer immunotherapy is influenced by the status of the TME, which regulates inflammation and immunoescape [3, 10, 41]. For example, hypoxic and inflammatory TME caused by antiangiogenic therapy has been targeted to improve the clinical outcome of cancer combination therapy, and several approaches were expected to enhance cancer immunotherapy by improving inflammation and increasing immune cell infiltration [14, 18, 42, 43]. The difficulty in vessel transportation causes the hypoxic and acidic TME (metabolic waste accumulation) to trigger inflammation and immunosuppression [44, 45], which is exacerbated by metabolic reprogramming into the glycolytic nature of cancer cells. That is, the collapsed vessels result in hypoxia in the tumor regions, which causes excess angiogenesis, metastasis, and metabolic reprogramming in the TME [10]. The highly aberrant angiogenesis and hypoxia contribute to the maintenance of immunosuppressive TME and cause cancer cells to resist immunotherapy [14]. In the TME, macrophages are abundant innate immune cells that play crucial roles in the cancer-immune response. The infiltrated macrophages (tumor-associated macrophage, TAM) are considered as the M2 phenotype, which provides an immunosuppressive tumor microenvironment. Important drivers of M2 phenotype are Th2 cytokines such as IL-4 and IL-13, the anti-inflammatory cytokines IL-10, and transforming growth factor-β (TGF-β). M2 macrophages also secrete a series of anti-inflammatory cytokines, which promote tumor growth, angiogenesis, and metastasis [46]. Furthermore, tumor-associated macrophage regulates the polarization of T cell to Treg and dendritic cell (DC) to DCreg and the differentiation of myeloid-derived suppressor cells (MDSC), which transforms into an immunosuppressive TME [47–49]. Taken together, vascular normalization is a promising approach that the treatment can inhibit extensive angiogenesis, and at the same time, the treatment can allow for eliminating thrombosis and improving inflammatory TME. Improved vessel perfusion could decrease hypoxia and further promote infiltration of immune cells and activate CD8 + T cells.

We indicated that the Fc-VFD inhibits the secretion of proangiogenic factor, VEGF-A and IL-6, from cancer cells (Fig. 4), endothelial cells (Fig. 5), and macrophages [2] in the TME. M2 macrophages secrete anti-inflammatory cytokines, such as IL-6, IL-10, IL-13, TGF-β, and chemokines and proteases, such as Arginase1, MMP, and VEGF-A, which promote tumor growth, angiogenesis, and metastasis [2, 46]. Since we found that Fc-VFD can suppress IL-6-induced angiogenesis, it is worth testing the inhibitory ability of Fc-VFD on the resistance to anti-VEGF-A therapies through other pro-angiogenesis cytokines. Another resistance mechanism of anti-angiogenesis is alternative cytokines released by different cells in the TME. For example, IL-10, IL-13, and TGF-β from cancer cells and macrophages induce angiogenesis [2]. In addition, IL-17, a pleiotropic proinflammatory cytokine mainly secreted by T-help 17 (Th17) cells gives impetus to angiogenesis [50, 51]. Many studies suggest that IL-17 secreted by stromal cells and recruited bone marrow-derived cells (BMDCs) together promotes the resistance to anti-angiogenesis therapy [44, 52, 53].

Our results found that the effect of Fc-VFD on the xenograft tumor model of HCT15 and B16/F10 cells did not follow a dose-dependent pattern at high concentrations (50–200 ng/kg), and it has the best inhibitory effect at a low-dose concentration of 37.5 ng/kg (Figs. 3C and 5A). Our findings indicate that a lower dose of Fc-VFD can promote vascular-normalizing ability and reprogram the tumor microenvironment away from immunosuppression toward activation of immunotherapy. In addition, lower-dose Fc-VFD antiangiogenic therapy is able to reduce drug resistance. The Fc-VFD is a chimeric dimer fusion protein of VEGF_121_ and VEGF_165_ that was connected by Fc regions of human IgG1 to enhance dimerization and immune cell targeting, which was confirmed by amino acid sequencing and tandem mass spectrometry [9]. A high concentration of Fc-VFD treatment may cause a “cytokine chain reaction” in the TME because the Fc-VFD we created is still a kind of active VEGF dimer with small modifications and deletion defects and binds on VEGFR1/2 of endothelial and cancer cells. It is possible that a high concentration of Fc-VFD treatment is similar to the effect of excess wild-type VEGF dimer on tumor cells and further induces other cytokine production. For instance, Fc-VFD can interact or activate another member of the VEGF family, PIGF. PIGF signaling activates inflammatory cytokine releasing, such as IL-1β and IL-6, to promote angiogenesis in several types of cancer [54, 55]. Other alternative proangiogenic or oncogenic pathways might be also activated by excess Fc-VFD in the TME [12, 18, 42]. The speculation should be proved by further analysis of gene expressing profiles from single-cell RNA sequencing of orthotopic-xenograft tumors. These findings are very similar to a previous study that suggests that appropriate lower-dose antiangiogenic therapy is an effective strategy to reengineer the tumor microenvironment for active immunotherapy [22]. Antiangiogenic agents generally are treated at relatively high doses (bevacizumab, 10 or 15 mg/kg body weight) in the treatment of breast cancers [56, 57]. The high-dose antiangiogenic therapy caused excessive trimming of tumor vessels, and it may exacerbate, rather than reverse, the immunosuppressive tumor microenvironment and thus may compromise the efficacy of active cancer immunotherapy. In addition, low-dose anti-VEGFR2 antibody treatment increased TAMs and decreased MDSCs in breast tumors [22]. Indeed, we found that improved vessel normalization decreases hypoxia and polarizes TAMs to an M1-like phenotype, and, in turn, elevated CD80/CD86 expression in M1-like and reduced MRC1/ARG1 expression in M2-like TAMs and further promotes T-cell tumor infiltration.

In recent years, combination therapies directed against different targets in the TME become mainstream of anti-tumor therapy. Given that the combinations of high doses of bevacizumab with chemotherapy have not improved the overall survival of cancer patients [57], a study suggests a vessel normalization strategy used in breast cancer is more effective with active combination immunotherapy [22]. Our in vivo results showed that the combination of Fc-VFD with ant-PD-L1 more effectively represses the tumor growth and stimulates vascular normalization in the mouse model. Our data also showed that Fc-VFD combined with Actemra (tocilizumab, anti-IL6R) or Avastin (Bevacizumab, anti-VEGF-A) are successful therapeutic strategies to normalize intratumor vessels and inhibit tumor growth. This combination is not harmful to mice according to the vitality and body weight. In the future, vascular normalization by Fc-VFD can combat the drug resistance to antiangiogenic therapy and combine with other types of cancer immunotherapy to reprogram the immunosuppressive TME and enhance the infiltrating ability of immune cells.

## Conclusion

The Fc-VFD fusion inhibits tumorigenesis through vascular normalization and reprogramming of TME that increases immune cells infiltration and represses M2 macrophages polarization. Importantly, Fc-VFD enhances the synergistic effect on the combination immunotherapy with anti-PD-L1 in vivo. In short, Fc-VFD normalizes intratumor vasculature to reprogram the immunosuppressive TME and enhance cancer immunotherapy.

## Supplementary Information

Below is the link to the electronic supplementary material.

Supplementary file1 (PDF 958 kb)

## Data Availability

Not applicable.

## Abbreviations

ANGPT1/2: Angiopoietin 1/2
CM: Conditioned medium
FBS: Fetal bovine serum
Fc-VFD: Fc-VEGF121-VEGF165 fusion antibody drug
HIF-1α: Hypoxia-inducible factor-1α
IHC: Immunohistochemistry
LPS: Lipopolysaccharide
NOS: Nitric oxide synthase
PD-L1: Programmed death-ligand 1
RTK: Receptor tyrosine kinase
TME: Tumor microenvironment
VEGF: Vascular endothelial growth factor
VEGF121: Vascular endothelial growth factor 121
VEGF165/A: Vascular endothelial growth factor 165/A
VEGFR2: VEGF receptor 2
