# Supplementary material for: A Fc-VEGF chimeric fusion enhances PD-L1 immunotherapy via inducing immune reprogramming and infiltration in the immunosuppressive tumor microenvironment
Source: Cancer Immunol Immunother. 2022 Jul 27;72(2):351–69. doi: 10.1007/s00262-022-03255-9 (PMC9870840; doi:10.1007/s00262-022-03255-9)
Supplement: Supplementary file 1 — Supplementary file1 (PDF 958 kb) [file 262_2022_3255_MOESM1_ESM.pdf]

A Fc-VEGF chimeric fusion enhances PD-L1 immunotherapy via inducing immune reprogramming and infiltration in the immunosuppressive tumor microenvironment

Cheng-Liang Kuo[1#], Han-Yu Chou[1#], Hui-Wen Lien[1,2], Chia-An Yeh[1,4], Jing-Rong Wang[2], Chung-Hsing Chen[1], Chi-Chen Fan[4,5], Chih-Ping Hsu[4], Ting-Yu Kao[4], Tai-Ming Ko[2,3], and Alan Yueh-Luen Lee[1,6,7]*

*: Corresponding author:

National Institute of Cancer Research, National Health Research Institutes, 35 Keyan Road, Zhunan, Miaoli 35053, Taiwan

Tel.: +886-37-206-166 ext. 31705

Fax: +886-37-586-463

E-mail: alanylee@nhri.edu.tw

ORCID ID: 0000-0003-0252-0571

Materials and methods

The primers of RT-PCR used in this study

Gene	Forward 5'-3'	Reverse 5'-3'
**hLon**	GTC ATG GAT GTT GTG GAC GA	GTA GTT GCG GGT GAC ATT GA
**hVEGFA**	AGG CCA GCA CAT AGG AGA GAT	CTT GTC ACA TCT TGC AAC GCG AG
**hIL-6**	TCA ATA TTA GAG TCT CAA CCC CCA A	GGA CCG AAG GCG CTT GT
**hCCL19**	GGT GCC TGC TGT AGT GTT CA	GGT CCT TCC TTC TGG TCC TC
**hARG1**	TGG ACA GAC TAG GAA TTG GCA	CCA GTC CGT CAA CAT CAA AAC T
**hMAF**	AGA GAC ACG TCC TGG AGT CG	GCT TCC AAA ATG TGG CGT AT
**hCCL13**	CTC CTC TGG CCT CCT CTT CT	ACC GAA TAC AAA CCC ACT GC
**hINOS**	ACA AGC CTA CCC CTC CAG AT	TCC CGT CAG TTG GTA GGT TC
**hCCR7**	ACA TCG GAG ACA ACA CCA CA	GGA AGG GTC AGG AGG AAG AG
**hCXCL11**	TCG AAG CAA GCA AGG CTT AT	GTC CTT TCA CCC ACC TTT CA
**hARG1**	TGG ACA GAC TAG GAA TTG GCA	CCA GTC CGT CAA CAT CAA AAC T
**hMRC1**	GGG TTG CTA TCA CTC TCT ATG C	TTT CTT GTC TGT TGC CGT AGT T
**mLon**	ATG ACC GTC CCG GAT GTG T	CCT CCA CGA TCT TGA TAA AGC G
**mIL-6**	AGT TGC CTT CTT GGG ACT GA	TCC ACG ATT TCC CAG AGA AC
**mVEGFA**	AAA GGC TTC AGT GTG GTC TGA GAG	GGT TGG AAC CGG CAT CTT TAT C
**mTGF-β1**	GTG TGG AGC AAC ATG TGG AAC TCT A	TTG GTT CAG CCA CTG CCG TA
**mIFNγ**	TGG CAT AGA TGT GGA AGA AAA GAG	TGC AGG ATT TTC ATG TCA CCA T
**mPD-L1**	GAC CAG CTT TTG AAG GGA AAT G	CTG GTT GAT TTT GCG GTA TGG
**mIL-10**	TGA ATT CCC TGG GTG AGA A	TGG CCT TGT AGA CAC CTT GG
**mANGPT1**	AAC CGA GCC TAC TCA CAG TAC G	GCA TCC TTC GTG CTG AAA TCG G
**mANGPT2**	AAC TCG CTC CTT CAG AAG CAG C	TTC CGC ACA GTC TCT GAA GGT G
**mS1PR1**	ATG GTG TCC ACT AGC ATC CC	CGA TGT TCA ACT TGC CTG TGT AG
**mNOS**	GAG ACA GGG AAG TCT GAA GCA C	CCA GCA GTA GTT GCT CCT CTT C
**mSTAT3**	CAA TAC CAT TGA CCT GCC GAT	GAG CGA CTC AAA CTG CCC T
**mCCR7**	TGT ACG AGT CGG TGT GCT TC	TAG GCC CAG AAG GGA AGA AT
**mARG1**	CTC CAA GCC AAA GTC CTT AGA G	AGG AGC TGT CAT TAG GGA

Cancer cell lines and cell culture

3B11 (*Mus musculus* axillary lymph node; vascular epithelium) cells were purchased from ATCC (#CRL-2160, Manassas, VA, USA) and cultured in DMEM (Dulbecco's Modified Eagle Medium). SW480 cells were cultured in L-15 (Leibovitz) medium with 20 mM glutamine and SW620 cells were cultured in L-15 medium. HCT15 (Human, colon, colorectal adenocarcinoma), HT29 cells, and THP1 (Human, monocyte, acute monocytic leukemia) were cultured in RPMI-1640 (Roswell Park Memorial Institute) medium. OEC-M1 cells, 293T (Human Embryonic Kidney cells 293), RAW264.7 (*Mus musculus* macrophage macrophage, Abelson murine leukemia virus-induced tumor), and B16/F10 mouse melanoma cancer cells were cultured in DMEM. All above mentioned culture medium were supplemented with 10% heat-inactivated fetal bovine serum (FBS), penicillin 100 Units/mL, streptomycin 100 μg/mL. All cell lines were carried out STR genotyping analysis and detection of mycoplasma by EZ-PCR kit (Biological Industries).

Purification of $VEGF_{121}$-$VEGF_{165}$ fusion proteins (VFD)

Purification of $VEGF_{121}$-$VEGF_{165}$ fusion proteins was performed as described previously [9]. The day before transfection, 293T cells were seeded in 15 cm dish with $3x10^6$ in 15 mL medium. The day of transfection, first, the medium were removed and replaced with 15 mL fresh medium. Second, the plasmid DNA ($VEGF_{121}$-$VEGF_{165}$, 15

μg) was transfected to 293T cells and replaced fresh medium after 6 hours. Then the medium were collected from the transfected 293T cells in 500 mL bottle with 10 mL syringes and filtered by the syringe filter. Next, the medium were added protein degradation inhibitors (cOmplete™, Mini, EDTA-free Protease Inhibitor Cocktail). Before purification, 4 mL NICKEL NTA Agarose Resin was premixed with binding buffer (50 mM NaH_2PO_4, 10 mM imidazole, 300 mM NaCl) in 15 mL tube and centrifuged at 1000 rpm for 5 minutes. Then per 45 mL conditioned medium were mixed with 1 mL NICKEL NTA Agarose Resin in binding buffer in 50 mL tube shaking overnight in cold room. Next, the Agarose Resin beads were packed into column tube. The beads were washed with 20 mL washing buffer (50 mM NaH_2PO_4, 20 mM imidazole, 300 mM NaCl). Then, the fusion proteins were eluted by 4 mL Elute Buffer (50 mM NaH_2PO_4, 250 mM imidazole, 300 mM NaCl) and were transferred into dialysis tubing (B/D Dialysis Tubing) for PBS buffer exchange. The fusion proteins were concentrated by centrifuge tube (Amicon ® ultracel 50 K) at 3000 rpm for 3 to 5 minutes. The concentrated protein was added by 10% glycerol and stored at -20 °C. According to the qualitative curve, the relative abundance of $VEGF_{121}$-$VEGF_{165}$ protein was determined.

Tube formation assay

An *in vitro* tube formation assay was employed by using 3B-11 endothelial cells that are

induced to invade a three-dimensional collagen gel where they form a network of capillary-like tubes. 3B-11 cell is a convenient endothelial cell model from mouse for tube formation assay [57]. Corning Matrigel® Matrix (BD Biosciences, San Jose, CA, USA) solution was thawed on ice overnight and 10 μl aliquots were coated onto a μ-slide angiogenesis (ibidi) and incubated at 37°C for 1h to solidify. 50 μl of DMEM supplemented with 10% FBS medium containing about $1x10^4$ endothelial cells were seeded onto the plated Matrigel Matrix. Medium contains Avastin, Actemra, or VFD and incubated the slide at 37°C for 2hr. The assay was done in triplicate and images of the formation of capillary-like structures were obtained with a computer-assisted microscope (Olympus, Tokyo, Japan) at 200x magnification. Tubular structures were quantified by manually counting the numbers of connected cells in randomly selected fields at 200x magnification.

Flow cytometry

THP-1 cells with different treatments were collected with Accutase Cell Detachment Solution (BD Biosciences) and resuspended at $1X10^6$ cells per ml in PBS containing diluted 1in 100 anti-mouse CD86 (PerCP-Cy™5.5) (BD, Pharmingen). For the identification of CD86 population, the samples were analyzed while using a FACSCalibur flow cytometer (BD Biosciences) and FlowJo 7.6 software.

Preparation for the single-cell RNA sequencing (scRNA-seq)

Tumors in the B16/F10 mouse melanoma were removed from subcutaneously injected mice and cell samples were prepared by the Tumor Dissociation Kit (Miltenyi Biotec). For excluding the influence of Red Blood cells and dead cells, Red Blood Cell Lysis (10X Genomic) and Dead Cell Removal Kit (Miltenyi Biotec) were applied and followed by the manufacturer's instructions. Single melanoma cells obtained from B16/F10 mice were loaded into a 10× Genomics microfluidics chip and encapsulated with barcoded hashtag-oligos (HTO)-containing gel beads using the 10× Genomics chromium controller (10X Genomics, Pleasanton, CA, USA). The single-cell suspension sample was loaded onto a chromium single cell chip, following the manufacturer's instructions for coencapsulation with barcoded gel beads. The captured mRNA was barcoded and then the barcoded cDNA was converted into pooled single-cell RNA-seq libraries for Illumina sequencing. After library preparation and sequencing of samples, we created the matrix of scRNA-seq by 10× Genomics Cell Ranger using software (version 3.1.0). Thereafter, we separated the matrix of scRNA-seq from B16/F10 mouse melanoma with different treatment using the cell hashing technique. This dataset was quality-controlled, and the negative cells were removed using the R package Seurat v3. For cell hashing, antibodies were added to TotalSeq-C0301, TotalSeq-C0302, TotalSeq-C0303, TotalSeq-C0304 and TotalSeq-C0305 anti-mouse MHC. The cells were

incubated for 30 min at 4 °C, followed by washing thrice in staining buffer. Libraries were constructed according to the manufacturer’s protocol, with the following modifications according to the CITE-Seq protocol (https://cite-seq.com/protocol).

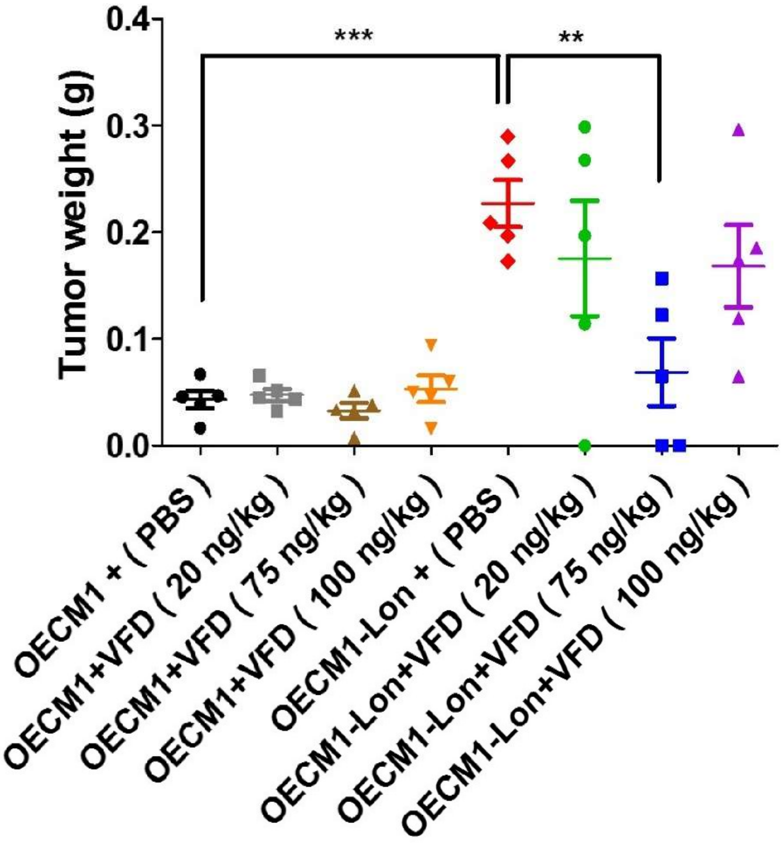

Supplemental figure 1

Figure S1. Fc-VFD efficiently inhibits Lon-induced tumorigenesis *in vivo.*

OECM1 cells overexpressing Lon or not were injected subcutaneously into BALB/C Nude mice using 1×10^6 cells in 100 µl. The mice bearing tumor were treated with different doses of Fc-VFD as indicated via intravenous injection (i.v.) at 11 days post inoculation for 3 times, once a week. The tumor weight was measured at the endpoint (44th day). Each dot represents one mouse. *p<0.05; **p<0.01.

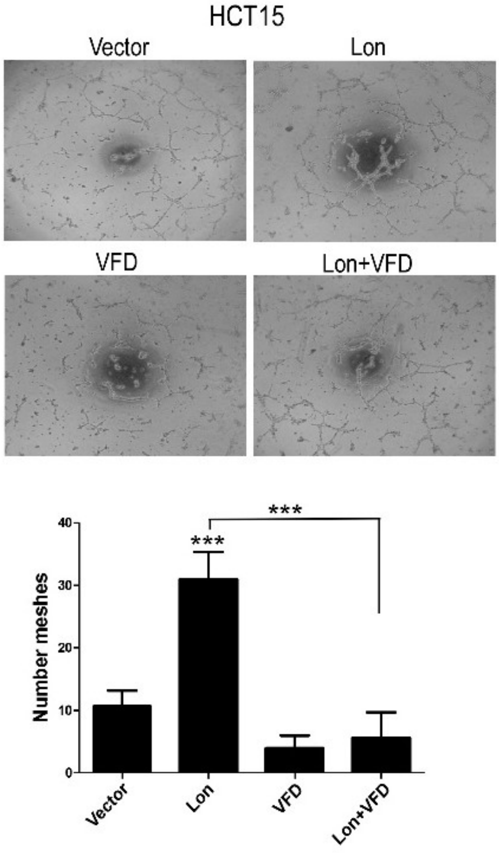

Figure S2. VFD inhibits angiogenesis induced by Lon overexpressing HCT15 cells. 3B-11 were cultured in HCT15 or HCT15 Lon overexpressing condition medium alone or treated with VFD (312.5μM). The tube formation was quantified by counting the connected cells at 200 X magnification (above panel) and the panels represent the standard deviation from three independent experiments (below panel).

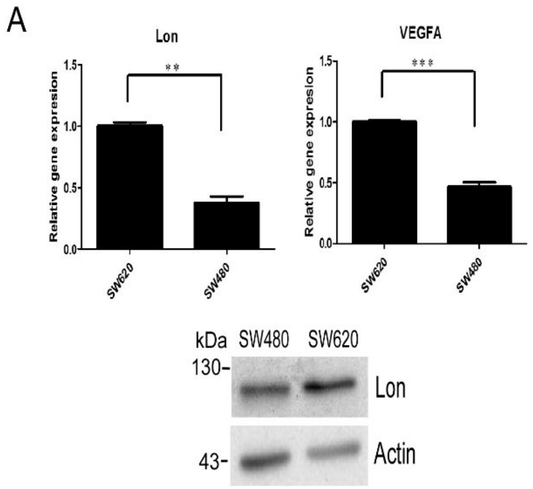

Figure S3. Endogenous Lon and VEGF-A expression levels in SW480 and SW620 colon cancer cells. The mRNA levels were analyzed by quantitative real-time PCR (top). Endogenous Lon protein expression was analyzed by Western blotting (bottom). The error bars represent the standard deviation from at least three independent experiments. **p<0.01; ***p < 0.001.

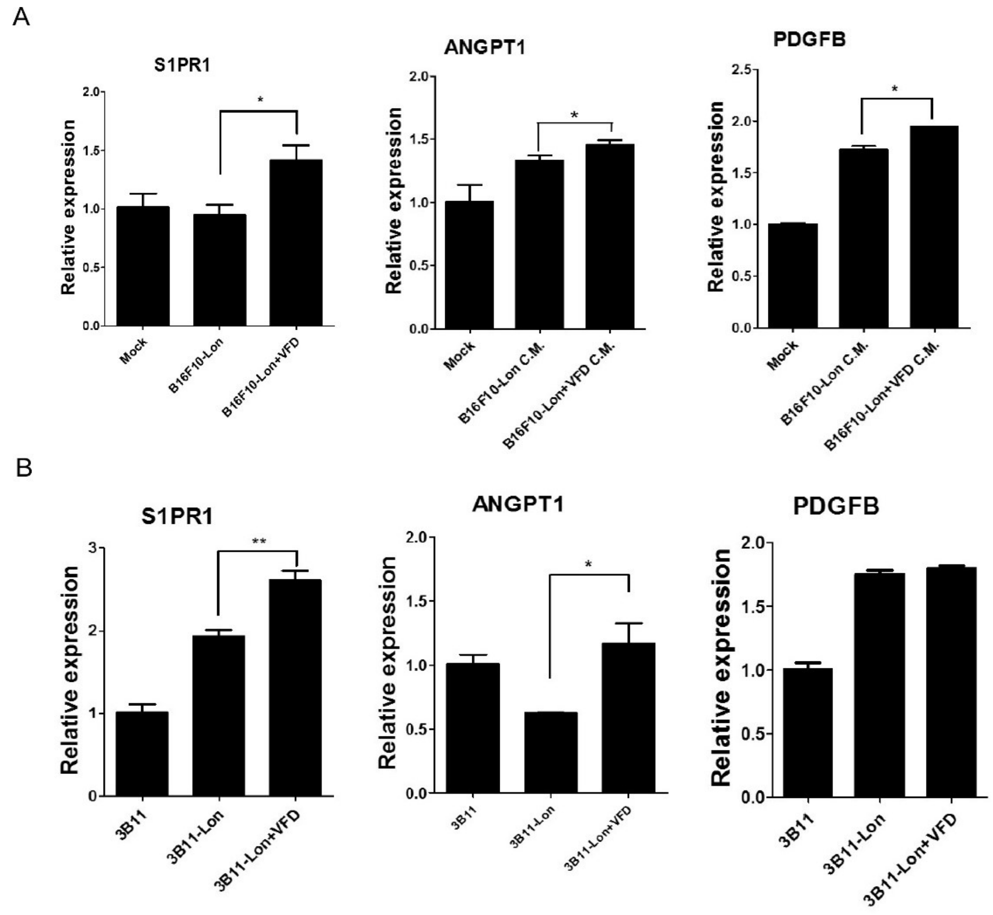

Supplemental figure 4

Figure S4. Fc-VFD induces Lon-inhibited anti-angiogenic factors in 3B11 endothelial cells

(A) The conditioned medium from B16/F10 cells transfected with Lon plasmid and treated with Fc-VFD (312.5 pM) or not for 24 hrs were collected. 3B-11 mouse endothelial cells were incubated with the conditioned medium. The mRNA expression in 3B-11 was analyzed by quantitative real-time PCR as indicated. The results are presented as fold increase related to the control cells (deliberately set to 1). Data are presented as mean ± SD of at least three independent experiments.

(B) 3B11 cells were transfected with the plasmids encoding Lon and treated with Fc-VFD (312.5 pM) or not for 24 hrs. The mRNA expression in 3B-11 was analyzed by quantitative real-time PCR as indicated. The results are presented as fold increase related to the control cells (deliberately set to 1). Data are presented as mean ± SD of at least three independent experiments. The error bars shown in the graphs represent the standard deviation from at least three different experiments. *p<0.05; **p<0.01, ***p < 0.001.

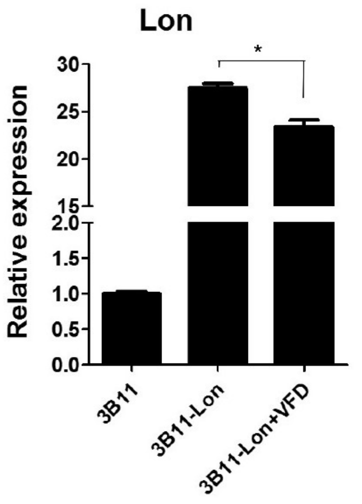

Figure S5. VFD inhibits Lon overexpression in 3B11 cells.

The mRNA expression of Lon was analyzed by quantitative real-time PCR. 3B-11 mouse endothelial cells were transfected with plasmid encoding Lon and treated with VFD (312.5μM). The panels represent the standard deviation from three independent experiments. *$p<0.05$; **$p<0.01$; ***$p < 0.001$.
